# Towards disentangling the classification of freshwater fish trypanosomes

**DOI:** 10.1007/s42995-023-00191-0

**Published:** 2023-10-13

**Authors:** Peng Zhang, Jie Liu, Xiao-Ming Yin, Jun-Yu Zhou, Julius Lukeš, Zhao-Rong Lun, De-Hua Lai

**Affiliations:** 1 Guangdong Provincial Key Laboratory of Aquatic Economic Animals, State Key Laboratory of Biocontrol, School of Life Sciences, Sun Yat-sen University, Guangzhou, 510275 China; 2 BGI Genomics, BGI-Shenzhen, Shenzhen, 518083 China; 3 Institute of Parasitology, Biology Centre, Czech Academy of Sciences, České Budějovice (Budweis), 37005 Czech Republic; 4 Biomedical Research Centre, School of Science, Engineering and Environment, University of Salford, Salford, M5 4WT UK

**Keywords:** Freshwater fish trypanosomes, 18S rRNA, Morphology, Phylogeny

## Abstract

Currently, new species of freshwater fish trypanosomes, which are economically important parasites, are being described based on subjectively selected features, i.e., their cell morphology and the host species. We have performed detailed phylogenetic and haplotype diversity analyses of all 18S rRNA genes available for freshwater fish trypanosomes, including the newly obtained sequences of *Trypanosoma carassii* and *Trypanosoma danilewskyi*. Based on a sequence similarity of 99.5%, we divide these trypanosomes into 15 operational taxonomic units, and propose three nominal scenarios for distinguishing *T. carassii* and other aquatic trypanosomes. We find evidences for the existence of a low number of freshwater fish trypanosomes, with *T. carassii* having the widest geographic and host ranges. Our analyses support the existence of an umbrella complex composed of *T. carassii* and two sister species.

**Supplementary Information:**

The online version contains supplementary material available at 10.1007/s42995-023-00191-0.

## Introduction

Trypanosomes are hemoflagellates that parasitize all groups of vertebrates, including marine and freshwater fishes (Kostygov et al. 2021), among which they are transmitted by blood-sucking leeches (Khan 1976; Letch 1980) or other blood-sucking invertebrates. Symptoms of fish trypanosomiases range from mild anemia to deadly infections with a high parasite burden (Dyková and Lom 1979; Khan 1985). Serious trypanosome infections were previously documented in a range of fish species, yet their economic impact was rather limited (Woo and Poynton 1998). This has changed with the development of a large-scale aquaculture industry, characterized by high density of fish associated with stress and easy transmission of pathogens. Indeed, several recently documented outbreaks of trypanosomiases in fishes (Jesus et al. 2018; Luo et al. 2019; Su et al. 2014) fulfill the criteria of newly emerging diseases with a potential of major economic losses. Largely due to the lack of our knowledge about the biology of these widespread yet so far rather overlooked pathogens, no available against the fish trypanosomiasis.

Using various criteria, over 280 species of trypanosomes have been described from fishes (Chen et al. 2022; Eiras et al. 2012; Gupta and Gupta 2012; Jesus et al. 2018; Lemos et al. 2015; Su et al. 2014; Woo and Poynton 1998), mostly from freshwater hosts. While the traditional taxonomy of fish trypanosomes was based on their morphology and host specificity (Burreson and Pratt 1972; Qadri 1962), it is now generally accepted that these criteria are largely unsuitable for trypanosomes, due to the low number of measurable features, the high variability of these features, pleomorphism, and widely varying host specificity (Jansen et al. 2017; Kostygov et al. 2021). Indeed, Lom (1979) noticed the necessity for a thorough revision of named species of fish trypanosomes, since he considered most of them as synonymous. Just as an example, *Trypanosoma mukasai* from *Haplochromis* spp. was suspected to be synonymous with *Trypanosoma tobeyi* from *Clarias angolensis* (Baker 1960), which was later confirmed by cross-infection experiments (Negm-Eldin 1998).

Consequently, several attempts have been made to use other criteria to delimit fish trypanosome species, such as agglutinability of surface lectins (Zajíček and Lukeš 1992), activities of selected enzymes (Zajíček and Pecková 1995), size of kinetoplast DNA minicircles (Jirků et al. 1995), and the composition of surface carbohydrates (Feng and Woo 1998). The limits of morphology-based identification, also encountered in the well-studied trypanosomes of humans and other mammals (Hoare 1972), were largely circumvented by the adoption of methods based on conserved gene sequences, primarily 18S rRNA (Gibson et al. 2017) and glyceraldehyde 3-phosphate dehydrogenase (GAPDH) (Hamilton and Stevens 2017). Moreover, cross-infection experiments revealed that some trypanosomes are confined to a single host species (Noyes et al. 2002), while others, such as *Trypanosoma cruzi*, have been encountered in more than 100 mammalian species (Jansen et al. 2017).

So far, the 18S rRNA-based classification has been applied in numerous protist lineages, e.g., Sessilida (Lu et al. 2023), while in case of fish trypanosomes only to European freshwater fish trypanosomes, revealing their clustering into three clades, the composition of which did not agree with the morphology-based classification (Gibson et al. 2005). While some species fell into a single clade (e.g., *Trypanosoma abramidis*), others appeared in two (e.g., *Trypanosoma tincae*) or even all three clades (*T. carassii*) (Gibson et al. 2005). The latter species is the best studied fish trypanosome (Lom and Dyková 1992) that may occur also on other continents (Zhang et al. 2022). However, due to the lack of comprehensive sequence datasets, the species definition of *T. carassii* remains unresolved.

Here, we have established the 18S rRNA, GAPDH and internal transcribed spacer 1 (ITS1) sequences of several *T. carassii* isolates. The information contained in these sequences allowed us not only to re-address the taxonomic conundrum of *T. carassii*, but also to propose criteria for species definition suitable for freshwater fish trypanosomes. This should facilitate further research of these increasingly important parasites.

## Results

### 18S rRNA-based phylogenetic analysis

**Fig. 1:**
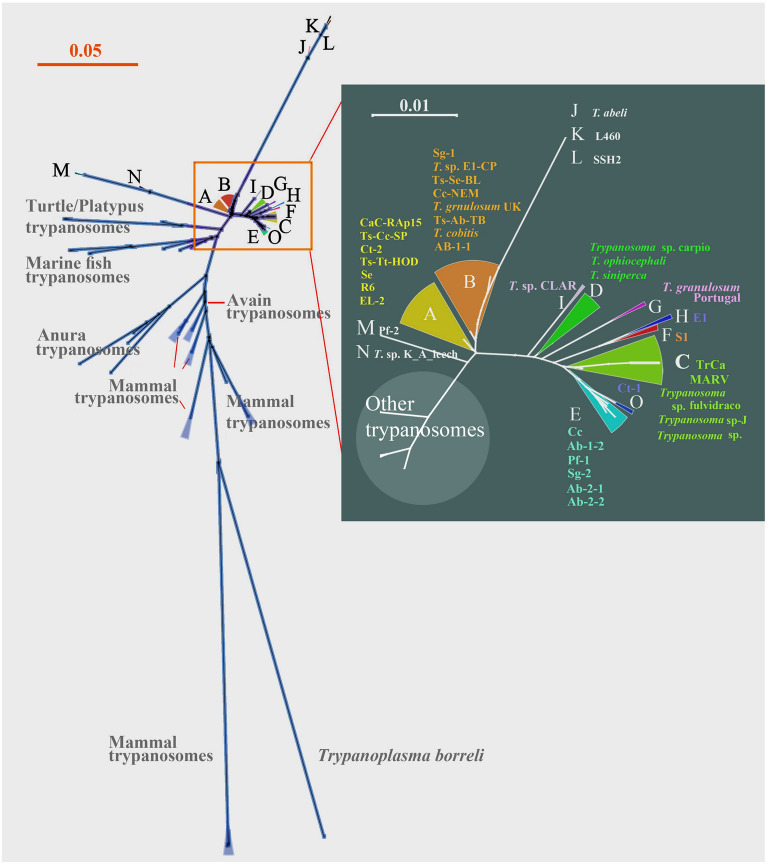
Phylogenetic analysis of 18S rRNA gene sequences from trypanosomes. 18S rRNA-based neighbor-joining phylogenetic tree of trypanosomes, bar represents 0.05 substitutions per site. Other trypanosomes (outgroup) are those of the marine clade and avian/mammalian species

First, we performed phylogenetic analysis using the 18S rRNA sequences of freshwater fish trypanosomes, as this gene is widely available and information-rich. Moreover, we generated four clones from a recently isolated *Trypanosoma* sp. micropteri that were found to be identical with the original sequence (OM397104) (Zhang et al. 2022). The *T. carassii* isolates described previously (Gibson et al. 2005; Zhang et al. 2022) and other trypanosomes related to *T. carassii* for which almost complete (> 2 kb) 18S rRNA sequences available were preferentially selected (Table 1). Phylogenetic trees were built using neighbor-joining and maximum likelihood methods. As expected, all freshwater fish trypanosomes group into a single clade, while marine fish trypanosomes form a sister group (Supplementary Fig. S1), similar to a merged tree (Fig. 1, merged from Supplementary Figs. S1–S6). However, within the freshwater clade, sequences could be subdivided into at least seven operational taxonomic units (OTUs). Among these, isolates EL-2, CaC-RA p15, R6, Ts-Cc-Sp and Se, Ts-Tt-HOD constitute OTU A, isolates Cc-NEM, Ts-Se-BL, Ts-AB-TB, LUMP 1243 and *T. granulosum* (UK) form OTU B, *T. danilewskyi* (TrCa), MARVp3, *Trypanosoma* sp. fulvidraco, *T. micropteri* (*T. danilewskyi*) and *Trypanosoma* sp. micropteri cluster into OTU C, and *Trypanosoma ophiocephali, T. siniperca* and *Trypanosoma* sp. carpio group together as OTU D. In addition, isolates Cc, CLAR (AJ620555 and OQ130038) and *Trypanosoma* sp. K_A from leech form OTUs E, I and N, respectively (Supplementary Fig. S1). These OTUs were built based on the genetic distances from MEGA analysis, with grouping criteria of ≤ 0.0040 (Supplementary Fig. S2), while the inter-group genetic distances are much larger, except A–B (0.0035–0.0071) on the edge of merge-or-split (Supplementary Table S1).

**Table 1:** Summarized information for trypanosomes used in this study

Isolate	Nominal trypanosome species	Host	Origin	Isolation date	GenBank access	Group
R6 clone1	*T. abramidis* Laveran & Mesnil, 1904	*Abramis brama*	Klesczewo, Poland	1979	AJ620554	A
Ts-Cc-SP clone 1	*T. carassii* Mitrophanow, 1883	*Cyprinus carpio*	South Bohemia, Czech Republic	1997	OQ130042	A
Ts-Tt-HOD clone 1	*T. tincae* Laveran & Mesnil, 1904	*Tinca tinca*	South Moravia, Czech Republic	1993	AJ620553	A
EL-2	*T. remaki*	*Esox lucius*	S. Bohemia, Czech Republic	1987	OQ130041	A
CaC-RA-p15	*T. carassii*	*Carassius carassius*	S. Bohemia, Czech Republic	1989	OQ130039	A
Se	*T*. sp. from *Scardinius erythrophthalmus*	*Scardinius erythrophthalmus*	Kyiv, Ukraine	2014	KJ601718	A
Ct-2	*T.* sp. from *C.* ‘*taenia’* (2)	*Cobitis taenia*	Kyiv, Ukraine	2014	KJ60720	A
Cc-NEM	*T. carassii*	*Cyprinus carpio*	S. Bohemia, Czech Republic	1992	OQ130040	B
LUMP 1243	*T*. *cobitis* Mitrophanow, 1883	*Nemacheilus barbatulus*	Essex, UK	1977	AJ009143	B
Ab-1–1	*T.* sp. from *Abramis brama* (1–1)	*Abramis brama*	Kyiv, Ukraine	2014	KJ601712	B
Sg-1	*T*. sp. from *Silurus glanis* (1)	*Silurus glanis*	Kyiv, Ukraine	2014	KJ601721	B
El-CP	*T. carassii* Mitrophanow, 1883	*Esox lucius*	S. Bohemia, Czech Republic	1990	L14841	B
Ts-Se-BL clone 1	*T. scardinii* Brumpt, 1906	*Scardinius erythrophtalmus*	Czech Republic	1987	AJ620550	B
*T. granulosum* clone 1	*T. granulosum* Laveran & Mesnil, 1909	*Anguilla anguilla*	Dorset, UK		AJ620551	B
Ts-Ab-TB clone 1	*T. abramidis* Laveran & Mesnil, 1904	*Abramis brama*	S. Bohemia, Czech Republic	1987	AJ620556	B
MARV clone 11	*T. carassii* Mitrophanow, 1883	*Cyprinus carpio*	S. Bohemia, Czech Republic		AJ620549	C
*T. danilewskyi*	*T. danilewskyi* Laveran and Mesnil 1904	*Carassius auratus gibelio*	S. Bohemia, Czech Republic	2004	AY527221	C
*T*. sp. fulvidraco	*T*. sp. fulvidraco	*Tachysurus fulvidraco*	Niushan Lake, China	2006	EF375883	C
*T. micropteri*	*T. micropteri*	*Micropterus salmoides*	Foshan City, China	2018	MH635421	C
*T*. sp. carpio	*T*. sp. carpio	*Cyprinus carpio*	Niushan Lake, China	2006	EF375882	D
*T. siniperca*	*T. siniperca*	*Siniperca chuatsi*	Taihu Lake, China	1964	DQ494415	D
*T. ophiocephali*	*T. ophiocephali*	*Channa argus*	Liao River, China	1964	EU185634	D
Ab-1-2	*T.* sp. from *A. brama* (1-2)	*Abramis brama*	Kyiv, Ukraine	2014	KJ601713	E
Cc	*T*. sp. from *Carassius carassius*	*Carassius carassius*	Kyiv, Ukraine	2014	KJ601715	E
Ab-2-1	*T*. sp. from *A. brama* (2-1)	*Abramis brama*	Kyiv, Ukraine	2014	KJ601716	E
Ab-2-2	*T*. sp. from *A. brama* (2-2)	*Abramis brama*	Kyiv, Ukraine	2014	KJ601717	E
Sg-2	*T.* sp. from *S. glanis* (2)	*Silurus glanis*	Kyiv, Ukraine	2014	KJ601722	E
Pf-1	*T*. sp. from *Perca fluviatilis* (1)	*Perca fluviatilis*	Kyiv, Ukraine	2014	KJ601724	E
Sl	*T*. sp. from *Sander lucioperca*	*Sander lucioperca*	Kyiv, Ukraine	2014	KJ601723	F
*T. granulosum* Portugal	*T. granulosum* Laveran & Mesnil, 1909	*Anguilla anguilla*	Portugal		AJ620552	G
El	*T.* sp. from *Esox lucius*	*Esox lucius*	Kyiv, Ukraine	2014	KJ601714	H
CLAR	*T*. sp. CLAR clone1	*Clarias angolensis*	Pet shop (import)	1999	AJ620555	I
CLAR-2	as above	as above	as above	as above	OQ130038	I
L4100	*T. abeli*	*Hypostomus luetkeni*	Minas Gerais, Brazil	2015	KR048310	J
L460	*T*. sp.	*Hypostomus luetkeni*	Minas Gerais, Brazil	2015	KR048306	K
SSH2	*T*. sp.	*Haementeria brasiliensis*	Minas Gerais, Brazil	2015	KR052820	L
Pf-2	*T*. sp. from *P. fluviatilis* (2)	*Perca fluviatilis*	Kyiv, Ukraine	2014	KJ601725	M
K&A	*T*. sp. K&A leech	*Piscicola geometra*	England	1977	AJ009167	N
Ct-1	*T*. sp. from *Cobitis ‘taenia’* (1)	*Cobitis taenia*	Kyiv, Ukraine	2014	KJ601719	O
	*T. chelodinae*	*Emydura signata*	Australia	2001	AF297086	Out group
	*T. binneyi*	*Ornithorhynchus anatinus*	Australia	1999	AJ132351	
	*T. pleuronectidium*	*Gadus morhua*	Norway	1999	DQ016613	
	*T. murmanensis*	*Hippoglossus hippoglossus*	Norway	2005	DQ016616	
	*T. boissoni*	*Zanobatus schoenleinii*		1973	U39580	
	*T. epinepheli*	*Epinephelus fuscoguttatus*	China	2013	JQ999962	
	*T. triglae*	*Trigla lineata*	Dakar Bay, Senegal	1967	U39584	
	*T. avium*				AJ009140	
	*T. lewisi*				AJ223566	
	*T. melophagium*				FN666409	
	*T. theileri*				AB007814	
	*T. mega*				AJ223567	
	*T. therezieni*				AJ223571	
	*T. rotatorium*				AJ009161	
	*T. neveulemairei*				AF119809	
	*T. cruzi*				AJ009147	
	*T. rangeli*				AJ009160	
	*T. dionisii*				AJ009151	
	*T. brucei*				M12676	
	*T. evansi*				AJ009154	
	*T. equiperdum*				AJ223564	
	*T. brucei gambiense*				AJ009141	
	*Trypanoplasma borreli*				L14840

To further verify consistency of this grouping, we analyzed the degree of DNA divergence among the above-characterized OTUs by DnaSP6.0. The average number of nucleotide differences (*K*) within each OTU ranged from 1 to 4.6000, while the nucleotide diversity (*Pi*) ranged from 0.0005 to 0.0023 (Table 2). The corresponding inter-group *Kxy* and *Dxy* were much larger (9.1670–89.4000 and 0.0045–0.0442) (Table 3).

**Table 2:** Average number of nucleotide difference (K) and nucleotide divergence (*Pi*) among intra-groups

	Average number of nucleotide differences (*K*)	Nucleotide diversity (*Pi*)
A	1.0000	0.0005
B	3.0670	0.0015
C	4.6000	0.0023
D	2.0000	0.0010

Groups E, I, N each contains only one isolate and are therefore not included

**Table 3:** Average number of nucleotide difference *Kxy* (above the diagonal) and nucleotide divergence *Dxy* (below the diagonal) among inter-groups

	A	B	C	D	E	I	N
A		9.1670	24.9000	20.8330	25.5000	27.5000	77.5000
B	0.0045		29.5330	24.6670	29.6670	28.5000	80.5000
C	0.0123	0.0146		18.0670	14.0000	29.6000	89.4000
D	0.0102	0.0121	0.0089		23.6670	25.3330	82.3330
E	0.0125	0.0146	0.0069	0.0116		29.0000	87.0000
I	0.0135	0.0140	0.0146	0.0124	0.0142	–	85.0000
N	0.0381	0.0396	0.0442	0.0405	0.0428	0.0419	–

–: Only one representative isolate in the group

When gaps are included, the genetic distances of these sequences display a pattern similar to that described above (Supplementary Fig. S2, Table S2). However, the genetic distances are significantly higher, particularly the intra-group distances within OTU C (≤ 0.0093), and exceed the inter-group distances for OTUs A and B (0.0044–0.0088), suggesting a merge of A and B into a single combined OTU. Unfortunately, we were not able to calculate *K*, *Pi*, *Kxy* and *Dxy* including the gaps.

In order to incorporate all relevant sequence data into the phylogenetic tree, we truncated the 2 kb-long alignment into a 1.4 kb-long version, so that it matched the length of 18S rRNA regions available for several fish trypanosomes in GenBank. Thus, following the integration of Ct-1, E1 and Sl, the above-described intra-group distances were adapted to ≤ 0.0015 (OTU A), ≤ 0.0067 (OTU B), ≤ 0.0051 (OTU C) and ≤ 0.0014 (OTU D). Consequently, the shortest inter-group distances are between OTUs A and B (0.0044–0.0114), and C and E (0.0059–0.0105), on the edge of merge-or-split (Supplementary Fig. S3, Table S3). The truncated distances within the intra-group *K* range from 0.6670 to 4.0000, and *Pi* ranges from 0.0005 to 0.0029 (Supplementary Table S4), while the corresponding inter-group *Kxy* and *Dxy* are much larger (9.4580–80.0000 and 0.0068–0.0578) (Supplementary Table S5). Moreover, when gaps are included, the genetic distances of these sequences display yet another pattern, with maximum intra-group genetic distances present in OTU C (≤ 0.0128), exceeding the inter-group distances of OTUs A and B (0.0058–0.0122) (Supplementary Table S6). Consequently, Ct-1 (KJ601719) stands out as a new OTU O, while E1 (KJ61714) and S1 (KJ61723) form OTUs H and F, respectively, with all three constituting sister groups of OTUs E and C (Supplementary Fig. S3).

Using the same strategy, we generated 18S rRNA datasets of various lengths (0.6, 1.2, and 1.5 kb), allowing us to place Ct-2 (KJ601720) in OTU A, Ab-1-2 (KJ601713) and Sg-2 (KJ601722) in OTU E, Pf2 (KJ601725) in OTU M, *T. granulosum* (Portugal) (AJ620552) in OTU G, *T. abeli* (KR048310) in OTU J, L460 (KR048306) in OTU K, and SSH2 in OTU L (Supplementary Figs. S4–S6, Tables S7–S18).

We were also able to incorporate all the above sequences into a larger dataset of various lengths and generate a new phylogenetic tree (Fig. 1). We found that all the defined 15 OTUs retain the same stable evolutionary relationships in the new phylogenetic tree, as those based on analyses of partial data (Supplementary Figs. S1–S6). In particular, OTUs A and B are on the edge of merge-or-split, while two groups (J/K/L and M/N) are divergent (Table 1).

**Fig. 2:**
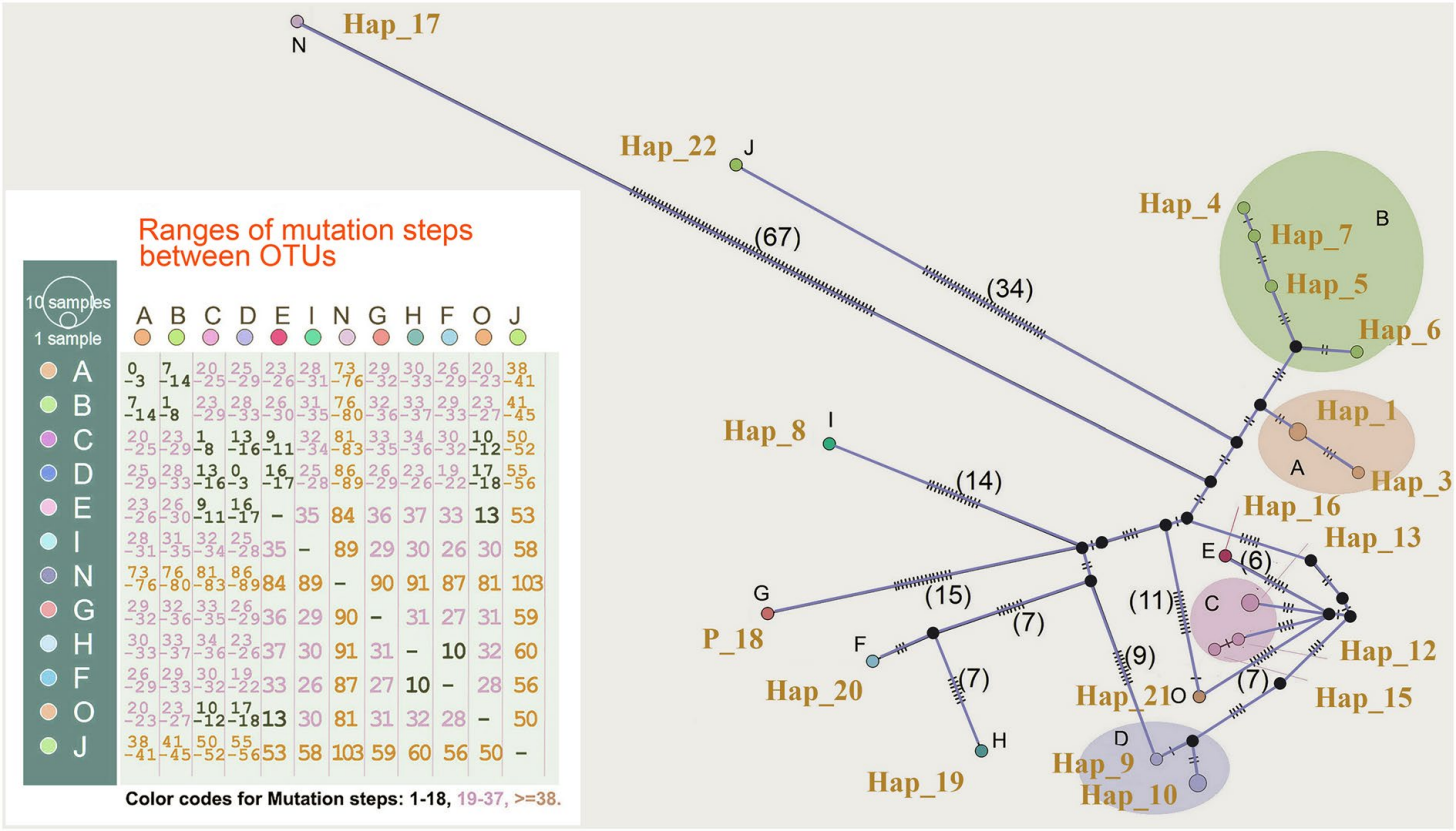
Parsimony network (TCS) of haplotype diversity based on 18S rRNA gene. The size of the circles is proportional to haplotype frequency and the colors represent the OTUs to which they belong. The mutation steps between each OTU pair are indicated

Next, we compared the divergence among each 18S rRNA dataset and established their correlation coefficients (Supplementary Fig. S2), which may allow data from various 18S rRNA regions to be correlated. For this, we generated Parsimony (TCS) and Neighbor-Net networks with missing data replaced by corresponding sites from full-length OTUs A (CaCRA-p15), C (MARV p3), D (EF375882), E (KJ601715) and I (AJ620555) in turn, with the corresponding mutation steps illustrated (Fig. 2; Supplementary Fig. S7). This approach confirmed that OTUs N and J, representing J/K/L and M/N, respectively, are significantly divergent from the remaining OTUs of the network. Although the ranges of mutation steps among 10 OTUs seem to overlap, the relationships within A/B, C/D/E/O and F/H, respectively, are very close (Fig. 2; Supplementary Fig. S8).

### GAPDH- and ITS1-based phylogenetic analyses

**Fig. 3:**
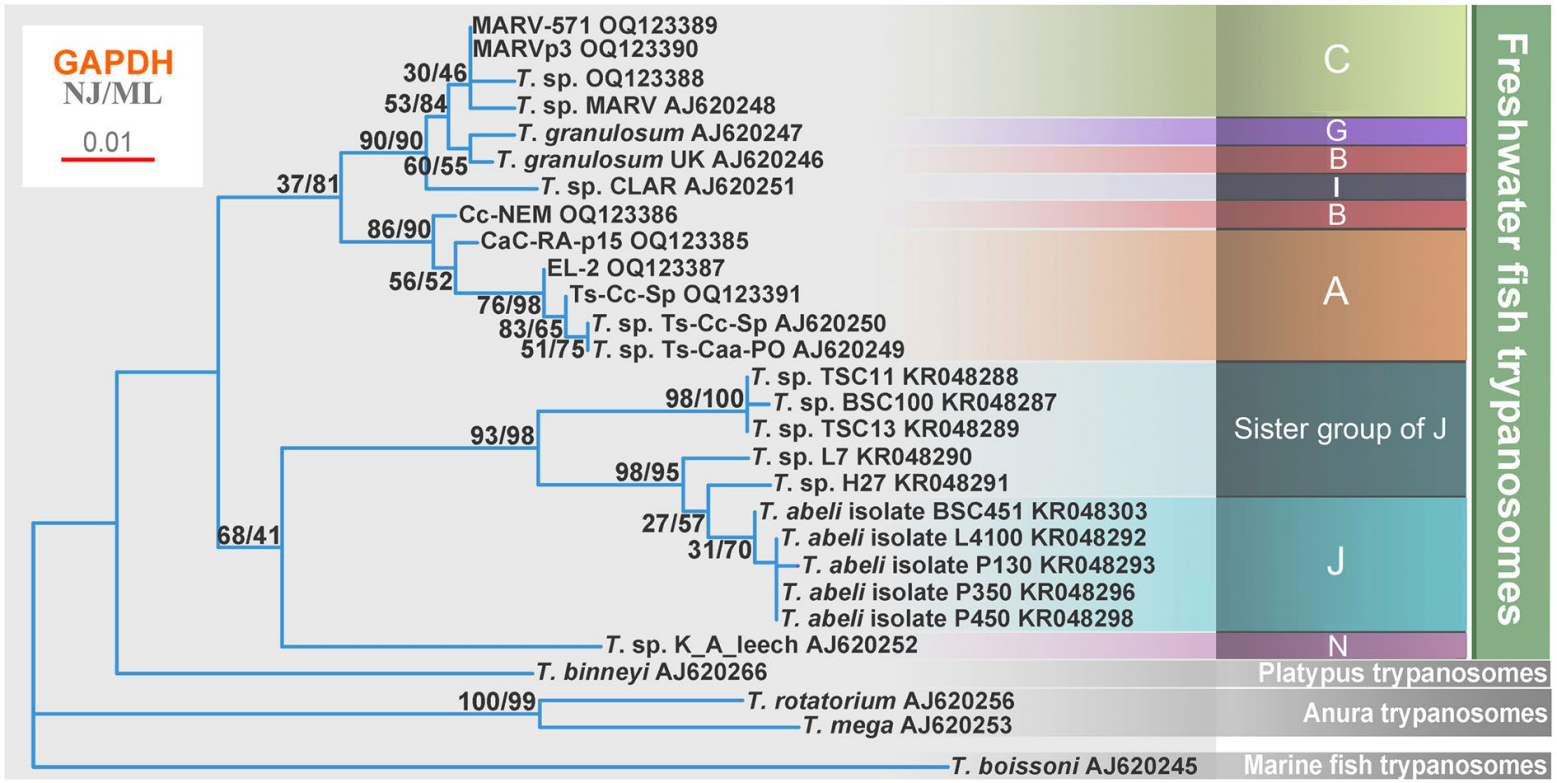
Phylogenetic analysis of GAPDH gene sequences from freshwater fish trypanosomes. GAPDH-based phylogenetic tree of trypanosomes. Bootstrap values (of neighbor-joining/maximum likelihood methods) shown at the nodes were counted with 1000 repetitions. Bar represents 0.01 substitutions per site

To increase the robustness of our phylogenetic study of freshwater fish trypanosomes, we also analyzed the GAPDH gene, which is generally the second marker of choice for reconstructing trypanosome phylogeny (Gibson et al. 2017; Hamilton and Stevens 2017). However, this gene sequence is available from a narrower set of species as compared to the 18S rRNA gene. Still, it allowed us to interrogate the relationships among seven OTUs. Consistent with the results obtained with the 18S rRNA sequences, the GAPDH sequences robustly split fish trypanosomes into a freshwater clade and a marine clade, with OTUs A grouping alone, and OTUs I and G grouping with C (Fig. 3). However, *T. granulosum* (UK) (AJ620246), which in the 18S rRNA-based tree groups with OTU B, affiliates in the GAPDH dataset with OTU G. At this point, however, we cannot rule out the possibility of miss-labeling, since some isolates of *T. granulosum* (e. g., Portugal) firmly group with OTU G (Fig. 3).

ITS1 is another useful marker for population structure within a species (Wen et al. 2016), although it has not previously been applied to fish trypanosomes. We generated 14 ITS1 sequences from EL-2 (*T. remaki*), CaC-RA p15 and Ts-Cc-Sp from OTU A, *T. carassii* (Cc-NEM) from OTU B, MARV p3 and *Trypanosoma* sp. micropteri from OTU C, and *Trypanosoma* sp. (CLAR) from OTU I. Our comprehensive analysis of this marker shows that ITS1 is highly divergent, with variability even within the same DNA sample. Nevertheless, the obtained data are consistent with OTUs A and B being indistinguishable, and OTUs I and C grouping together (Supplementary Fig. S9).

### Comparison of morphological parameters

**Fig. 4:**
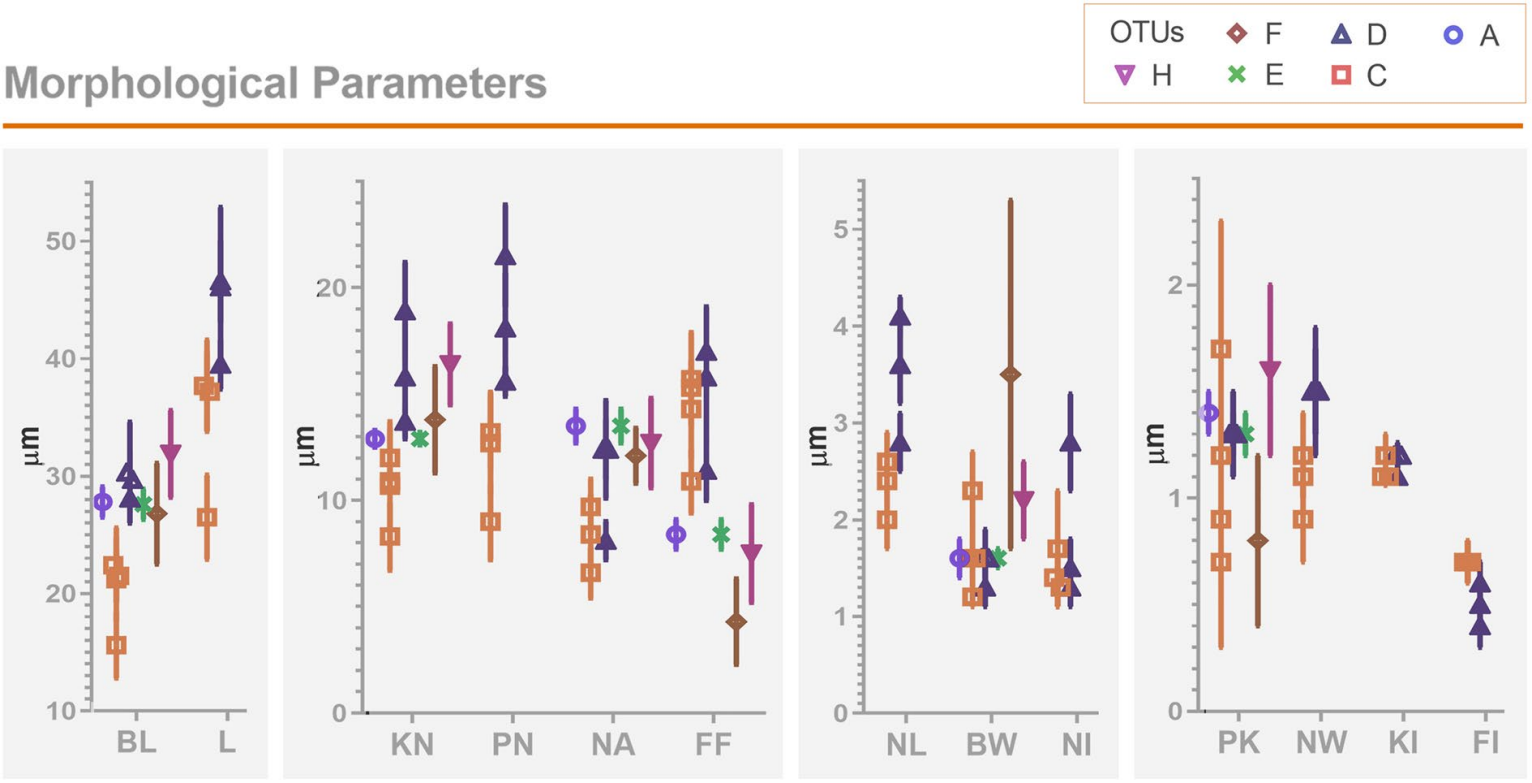
Morphological parameters of trypanosomes from different OTUs. *PK* posterior end to kinetoplast; *KN* kinetoplast to nucleus center; *PN* posterior end to nucleus center; *NA* nucleus center to anterior end; *BL* body length; *FF* free flagellum length; *L* total length including FF; *NL* nucleus length; *NW* nucleus width; *BW* body width, nuclear index NI = PN/NA, kinetoplast index KI = PN/KN, flagellar index FI = FF/BL

Next, we checked whether the published morphological data of freshwater fish trypanosomes provide any clue as to their taxonomy and phylogeny. To that end, only isolates with known molecular barcode(s) (e.g., 18S rRNA) were selected. In total, only one morphological dataset is available for OTUs A, E, F and H, while four datasets are available for OTU C and three for OTU D (Table 4). For members of OTUs C and D, the following parameters are available in the literature (distances in μm): PK (posterior end to kinetoplast), 0.7–1.7 and 1.3; KN (kinetoplast to nucleus), 8.3–12 and 13.7–18.9; PN (posterior end to nucleus), 8.4–12.7 and 15.6–21.5; NA (nucleus to anterior end), 6.6–9.7 and 8.1–12.4; BL (body length), 15.6–22.4 and 28.1–30.3; FF (free flagellum length), 10.9–15.7 and 11.4–17; L (total length including FF), 26.5–37.7 and 39.5–46.6, BW (body width), 1.2–2.3 and 1.3–1.6, respectively (Table 4; Fig. 4). *Z*-test analysis on these parameters showed some significant intra-OTU differences, e.g., L, NA, NL. However, a consistent inter-groups difference between OTUs C and D was not observed.

**Table 4:** Morphometrics of freshwater fish trypanosomes

Group and isolate		PK	KN	PN	NA	BL	FF	L
A	Se	1.4 ± 0.1	12.9 ± 0.4		13.5 ± 0.8		8.4 ± 0.7	27.8 ± 1.3
		1.1–1.5	10.2–13.6		9.6–14.9		4.5–11.4	22.9–30.0
C	*T.* sp.	1.2 ± 0.2	12.0 ± 1.7	8.4 ± 1.9	8.4 ± 1.9	21.5 ± 2.2	15.7 ± 2.2	37.2 ± 3.4
		0.8–1.9	8.0–16.1	3.9–13.0	3.9–13.0	17.0–26.6	8.5–22.3	26.2–46.7
C	*T.* sp. fulvidraco	0.9 ± 0.1	10.9 ± 2.3	12.7 ± 2.4	9.7 ± 1.3	22.4 ± 3.2	15.3 ± 0.9	37.7 ± 3.9
		0.7–1.1	6.0–14.3	7.3–16.2	8.0–11.2	15.5–26.4	12.5–15.8	28.7–42.0
C	*T. danilewskyi*	1.7 ± 0.6	10.7 ± 2.7			21.2 ± 3.64	14.3 ± 2.30	
		0.6–2.5	7.8–15.0			15.6–24.9	9.2–18.2	
C	*T.* sp.	0.7 ± 0.4	8.3 ± 1.6	9 ± 1.76	6.6 ± 1.24	15.6 ± 2.8	10.9 ± 1.5	26.5 ± 3.6
		0.1–2.1	3.7–12.4	4.5–13.4	3.5–9.5	8.9–21.4	4.2–15.2	14.2–33.9
D	*T.* sp. carpio	1.3 ± 0.2	18.9 ± 2.3	21.5 ± 2.4	8.1 ± 0.9	29.7 ± 2.5	17.0 ± 2.1	46.6 ± 3.5
		1.1–1.6	14.0–22.8	15.5–23.5	5.6–10.0	24.5–32.5	15.0–22.4	40.3–52.7
D	*T. siniperca*	1.3 ± 0.1	13.7 ± 0.8	15.6 ± 0.7	12.4 ± 1.4	28.1 ± 1.2	11.4 ± 1.4	39.5 ± 2.1
		1.2–1.7	12.5–15.9	14.3–17.6	10.8–16.4	27.2–34.6	10.3–14.9	36.7–45.3
D	*T. ophiocephali*	1.3 ± 0.2	15.8 ± 2.6	18.1 ± 2.6	12.4 ± 2.3	30.3 ± 4.3	15.8 ± 3	46.1 ± 6.8
		1.1–2.1	11.1–23.6	13.3–25.8	8.5–17.9	21.9–40.9	10.1–21.1	35.5–53.7
E	Cc	1.3 ± 0.1	12.9 ± 0.3		13.5 ± 0.8		8.4 ± 0.7	27.6 ± 1.3
		1.1–1.5	7.5–15.4		11.9–15.0		7.7–22.0	20.7–31.5
F	Sl	0.8 ± 0.4	13.8 ± 2.5		12.1 ± 1.3		4.3 ± 2.0	26.8 ± 4.3
		0.3–1.5	11.0–16.9		7.1–15.4		0–10.4	18.4–33.8
H	El	1.6 ± 0.4	16.4 ± 1.9		12.7 ± 2.1		7.5 ± 2.3	31.9 ± 3.7
		1.2–1.8	13.9–20.5		8.9–18.4		2.8–14.0	28.7–39.7

Group and isolate		NL	NW	BW	NI	KI	FI	*n* = ?	References
A	Se			1.6 ± 0.2			0.4*	132	Grybchuk-Ieremenko et al. (2014)
				1.4–1.8					
C	*T.* sp.	2.6 ± 0.3	1.8 ± 0.2 /1.2#	1.6 ± 0.2	1.7 ± 0.6	1.1 ± 0.02	0.7*		Jiang et al. (2019)
		1.9–3.3	0.7–0.9 /0.7–1.8	1.1–2.2	0.7–3.8	1.1–1.2			
C	*T.* sp. fulvidraco	2.4 ± 0.2	1.1 ± 0.1	1.2 ± 0.1	1.3 ± 0.2	1.2 ± 0.05	0.7*	80	Gu et al. (2007b)
		2.1–2.8	0.9–1.2	1.1–1.3	0.8–1.9	1.1–1.3	0.6–1.0*		
C	*T. danilewskyi*			2.3 ± 0.4				50	Woo (1981)
				1.6–3.1			0.7*		
C	*T.* sp.	2.0 ± 0.3	0.9 ± 0.16	1.6 ± 0.3	1.4 ± 0.2	1.1 ± 0.04	0.7 ± 0.1	217	Zhang et al. (2022)
		1.3–2.9	0.4–1.6	0.9–2.7	0.8–2.0	1.0–1.3	0.4–1.6		
D	*T.* sp. carpio	2.8 ± 0.3	1.5 ± 0.3	1.4 ± 0.3	2.8 ± 0.5	1.2 ± 0.06	0.6*	80	Gu et al. (2007b)
		2.5–3.8	1.1–2.3	/1.6# 1.3–2.1	2.0–4.3	1.1–1.4	0.5–0.7*		
D	*T. siniperca*	4.1 ± 0.2	1.5 ± 0.2	1.6 ± 0.1	1.3 ± 0.2	1.1 ± 0.02	0.3*	80	Gu et al. (2007a)
		3.7–4.3	1.2–1.8	1.3–2	0.9–1.5	1.1–1.2	0.2–0.3*		
D	*T. ophiocephali*	3.6 ± 0.4	1.2 ± 0.2	1.3 ± 0.2	1.5 ± 0.3	1.2 ± 0.03	0.3*	80	Gu et al. (2006)
		3.1–4.2	0.9–1.4	1.0–1.7	1.1–2.1	1.0–1.2	0.3–0.4*		
E	Cc			1.6 ± 0.1			0.4*	332	Grybchuk-Ieremenko et al. (2014)
				1.4–1.7					
F	Sl			3.5 ± 1.8			0.2*	28	Grybchuk-Ieremenko et al. (2014)
				1.5–5.5					
H	El			2.2 ± 0.4			0.3*	76	Grybchuk-Ieremenko et al. (2014)
				1.4–3.9					

Biometric data (center to center distances across the cell axis) in μm are provided as mean ± SD and ranges: *PK* posterior end to kinetoplast; *KN* kinetoplast to nucleus; *PN* posterior end to nucleus; *NA* nucleus to anterior end; *BL* body length; *FF* free flagellum; *L* total length; *NL* nucleus length; *NW* nucleus width; *BW* body width; *NI* nucleus index (PN/NA); *KI* kinetoplast index (PN/KN); *FI* flagellum index (FF/BL). #, two sets data were found from the published data. *, recalculated with the provided means of biometric data according to the definition

## Discussion

Although morphology was for almost a century the guiding principle of trypanosomatid taxonomy in general and fish trypanosomes in particular, for these morphologically highly variable flagellates this approach eventually became even less useful than for other protist groups (Letch 1979; Maslov et al. 2013). However, the application of molecular characters, now widely applied in the field of trypanosome research, turned out to be transformative. Here, we generated new sequences, and analyzed previously available sequence data, with the aim of reevaluating the taxonomy and systematics of freshwater fish trypanosomes that were so far rather neglected in this respect.

To avoid mixed infections (Grybchuk-Ieremenko et al. 2014), DNA was obtained either from clonal populations (Su et al. 2022) or even from single cells (Chen et al. 2022). When trying to analyze as many fish trypanosomes as possible, we encountered the problem of their frequent unavailability for DNA isolation. Another limiting aspect was that for numerous isolates, only short fragments of the 18S rRNA gene are available in GenBank. Indeed, fragments of the 18S rRNA gene shorter than 1.4 kb (Grybchuk-Ieremenko et al. 2014), or lacking the V7 and V8 domains, turned out to be unsuitable for phylogenetic analyses (Supplementary Fig. S2). One way of dealing with this problem is to predict the missing regions, allowing at least limited inferences for the corresponding trypanosome species.

Taking into consideration the varying length of sequences and their sometimes questionable quality, we decided to analyze only sequences with gaps excluded. Based on the 18S rRNA gene, all freshwater fish trypanosomes for which sequences are available fall into the following four major clades: (i) OTU A/B, (ii) OTU C-clade (I, D, G, E, O, C, F and H), and more distant, (iii) J/K/L, and (iv) M/N. This subdivision revealed that the *T. carassii* isolates TrCa and MARV (in OTU C) are not closely related to other *T. carassii* isolates (in OTU A/B), underlining the need for a reevaluation of the taxonomy of these trypanosomes (Fig. 1).

First of all, OTUs J/K/L and M/N clearly represent two distinct species, while the picture is more complex for the other 10 OTUs. The latter can be split into *T. carassii* (OTUs A/B) and eight distinct species (= scenario 1) or, following a more conservative approach, this group would be composed of *T. carassii* (OTUs A/B, syn. *Trypanosoma remaki, Trypanosoma scardinii, Trypanosoma abramidis*), *T. danilewskyi* (OTUs C/D/E/O), *T. granulosum* (OTU G) (with *T. granulosum* isolate UK transferred into *T. carassii*), and three unnamed trypanosomes (OTUs F/H, I and G) (= scenario 2). Another possibility is to lump all ten OTUs together into a single large *T. carassii* complex (= scenario 3).

Scenario 1 is based on genetic distances among full-size 18S rRNAs, excluding gaps, with intra-species differences below 0.4% and inter-species differences exceeding 0.6%. Very similar criteria have been applied in the case of trypanosomes of birds (Šlapeta et al. 2016). In the frame of this scenario, we suggest retaining TrCa as *T. danilewskyi* (syn. *Trypanosoma micropteri*) (Bienek et al. 2002), with the inclusion of other OTU C members, e. g., the MARV isolate. This would resolve a confusing situation associated with synonymizing *T. carassii* and *T. danilewskyi* (Lom and Dyková 1992), and numerous studies on *T. carassii* which actually used the *T. danilewskyi* isolates (Hagen et al. 2014; Islam and Woo 1991; Kovacevic et al. 2015; Wang and Belosevic 1994). Furthermore, *T. granulosum* shall be retained for OTU G, with OTU D representing *T. ophiocephali* (syn. *T. siniperca*), and OTUs E, O, I, H and F representing Cc, Ct1, CLAR, E1 and S1, respectively, all qualifying as separate species.

In scenario 2, we applied criteria corresponding to 3% differences in the 300 nt-long hypervariable V7 region (Smit et al. 2020) and 1% differences in the 1.4 kb-long region (Díaz et al. 2020), namely (excluding the gaps criteria of full-length 18S rRNA) intra-species distances less than 0.8%, and inter-species distances over 1.0%. Under these criteria, *T. danilewskyi* expands to include *T. ophiocephali* and *T. siniperca* (OTU D), as well as the unnamed trypanosomes from OTUs E and O, with *T. granulosum* restricted to isolate Portugal (OTU G), while OTUs F/H and I appear to be very closely related to African freshwater fish trypanosomes (only ~ 300 bp sequences available), which is potentially *T. mukasai* (Smit et al. 2020).

Finally, scenario 3 operates with excluded gaps, intra-species distances below 1.8% and inter-species distances above 4.0%. Such genetic distances are similar to those among members of the *Trypanosoma cruzi* complex, known to be highly divergent (Zingales et al. 2009), and *Trypanosoma rangeli* (Stevens et al. 1999). If such relaxed parameters were applied, all freshwater fish trypanosomes (10 OTUs) would belong to a single species—*T. carassii*.

Such a view finds some support in the highly divergent and thus problematic ITS1 data (Supplementary Fig. S9). Indeed, the differences in ITS1 among *T. cruzi* strains and among freshwater fish trypanosomes are very similar (Lima et al. 2015; Marcili et al. 2009).

Moreover, documented host ranges also support the last scenario. The overlap among OTUs A, B and C in the orders of fish hosts suggests the potential of different hemoflagellate isolates to infect the same fish species (Supplementary Fig. S10; Table 1) and, at the same time, the broad range of hosts firmly excludes the one-host one-trypanosome species paradigm.

This conclusion finds additional support in the morphology-based literature, which reports significant variability in the size and shape of these protists in the course of their life cycle, as well as depending on the intensity of the infection (Becker 1967; Gupta et al. 2006; Letch 1979; Mackerras and Mackerras 1961; Qadri 1962). Moreover, although there is a clear phylogenetic boundary between marine and freshwater fish trypanosomes, they may overlap in the estuarine environment, as was shown by artificial infections of the euryhaline tilapia (Chen et al. 2022). Therefore, the habitat-specific phylogeny may reflect an independent evolution of invertebrate vectors, such as leeches.

In conclusion, by interpreting all sequence data available for freshwater fish trypanosomes, we provide three alternative scenarios for their taxonomy. While species definition remains, unsurprisingly, ambiguous, we find best support for the existence of three robust species of freshwater fish trypanosomes, namely the umbrella complex of *Trypanosoma carassii*, *Trypanosoma abeli* (OTUs J/K/L) and yet-to-be named *Trypanosoma* sp. (OTUs M/N).

## Materials and methods

### DNA samples, PCR amplification and sequencing

Total DNA was extracted from the following freshwater fish trypanosomes: *Trypanosoma remaki* (isolate EL-2); *T. carassii* (CaC-RA p15, Ts-Cc-Sp, MARV p3, Cc-NEM) (Gibson et al. 2005); *Trypanosoma* sp. (CLAR) (Gibson et al. 2005); *T. danilewskyi* (TrCa) (Bienek et al. 2002; Kovacevic and Belosevic 2015; Woo 1981). All these strains are available in the isolate collection of the Institute of Parasitology, Czech Academy of Sciences. In addition, DNA was extracted from a clonal trypanosome parasitizing *Micropterus salmoides*, stored at the Sun-Yat sen University (Chen et al. 2022; Su et al. 2022).

The target regions were amplified using the following primers: 18S rRNA (5′-GACTTTTGCTTCCTCTATTG-3′, 5′-CATATGCTTGTTTCAAGGAC-3′), GAPDH (5′-GTGAAGGCGCAGCGCAAC-3′, 5′-CCGAGGATGYCCTTCATG-3′), and ITS1 (5′-CTGGATCATTTTCCGATG-3′, 5′-TGATACCACTTATCGCATT-3′). PCR reactions were conducted using the Phanta Super-Fidelity DNA Polymerase (Vazyme Biotech, China) according to the manufacturer’s protocol. PCR cycling parameters were as follows: initial denaturation at 94 °C for 3 min followed by 35 cycles at 95 °C for 15 s, 61 °C for 15 s, 72 °C for 2 min, and a final extension at 72 °C for 5 min, and the PCR products were sequenced (Thermo Fisher Scientific).

### Phylogenetic analysis

The obtained sequences were compared with the publicly available sequences using BLAST (http://www.ncbi.nlm.nih.gov/blast/). The 18S rRNA sequences, accession numbers and others information (Table 1) for freshwater fish trypanosomes were obtained from the GenBank database. Sequences were aligned by Clustal X (Thompson et al. 1997) using default settings and with final manual adjustments. The neighbor-joining and maximum likelihood methods were used to create phylogenetic trees by MEGA VII (Kumar et al. 2016) under Kimura's two-parameter model with gamma distributed and invariant sites (G + I), pairwise deletion for gaps and bootstrap of 1000 replicates.

Genetic distances between the newly obtained and available 18S rRNAs of freshwater fish trypanosomes were calculated by MEGA-VII and BLAST^+^ 2.8.1 using the following formula: genetic distances = 1 − the number of bases that can be paired between two sequences/the part of the alignment of the two sequences (1 − sequence identity).

DnaSP v6 software was used to calculate the average number of nucleotide differences (*Pi*) within a population (*K*), the average number of nucleotide differences among populations (*Kxy*) and the degree of nucleotide ambiguity (*Dxy*, *p*-distance). Significance analysis of morphological data was performed with *Z*-test (Zhang et al. 2022).

Parsimony (TCS) and neighbor networks of haplotype diversity were generated using DnaSP, PopArt and SplitsTree software, with missing data replaced in rotation by corresponding sites from full-length (> 2 kb) operational taxonomic units (OTUs) (Supplementary Fig. S11).

### Supplementary Information

Below is the link to the electronic supplementary material.

Supplementary file1 (DOCX 2206 KB)

## Data Availability

The datasets presented in this study can be found in online repositories. The accession numbers can be found at: https://www.ncbi.nlm.nih.gov/genbank/ (OQ130038, OQ130039, OQ130040, OQ130041 and OQ130042).
