# Supplementary material for: Towards disentangling the classification of freshwater fish trypanosomes
Source: Mar Life Sci Technol. 2023 Oct 13;5(4):551–63. doi: 10.1007/s42995-023-00191-0 (PMC10689643; doi:10.1007/s42995-023-00191-0)
Supplement: Supplementary file 1 — Supplementary file1 (DOCX 2206 KB) [file 42995_2023_191_MOESM1_ESM.docx]

**Supplementary materials**

**Towards disentangling the classification of freshwater fish trypanosomes**

**Peng Zhang^1^, Jie Liu^2^, Xiao-Ming** **Yin^1^, Jun-Yu Zhou^1^, Julius Lukeš^3^, Zhao-Rong Lun^1,4^, De-Hua Lai^1*^**

^1^Guangdong Provincial Key Laboratory of Aquatic Economic Animals, State Key Laboratory of Biocontrol, School of Life Sciences, Sun Yat-sen University, Guangzhou 510275, China;

^2^BGI Genomics, BGI-Shenzhen, Shenzhen 518083, China;

^3^Institute of Parasitology, Biology Centre, Czech Academy of Sciences and Faculty of Science, University of South Bohemia, České Budĕjovice (Budweis) 37005, Czech Republic;

^4^Biomedical Research Centre, School of Science, Engineering and Environment, University of Salford, Salford M5 4WT, United Kingdom

* Corresponding author: De-Hua Lai, Guangdong Provincial Key Laboratory of Aquatic Economic Animals, State Key Laboratory of Biocontrol, Sun Yat-sen University, Guangzhou 510275, China.

Tel: 86-020-84113010; Email: [laidehua@mail.sysu.edu.cn](mailto:laidehua@mail.sysu.edu.cn)

**Table S1. Genetic distances of 18S rRNA between 23 freshwater fish trypanosomes, excluding gaps.**

|  | A | B | C | D | E | I | N |
| --- | --- | --- | --- | --- | --- | --- | --- |
| A | 0.0005±0.0007  0-0.0015 |  |  |  |  |  |  |
| B | 0.0046±0.0009  0.0035-0.0071 | 0.0015±0.0012  0-0.0040 |  |  |  |  |  |
| C | 0.0136±0.0012  0.0118-0.0156 | 0.016±0.0012  0.0134-0.0178 | 0.0025±0.0019  0-0.0040 |  |  |  |  |
| D | 0.0107±0.0006  0.0102-0.0118 | 0.0127±0.0010  0.0107-0.0140 | 0.0103±0.0014  0.0075-0.0117 | 0.0020±0.0014  0-0.0030 |  |  |  |
| E | 0.0133±0.0006  0.0129-0.0145 | 0.0156±0.0007  0.0145-0.0167 | 0.0078±0.0017  0.0060-0.0091 | 0.0130±0.0005  0.0123-0.0134 | - |  |  |
| I | 0.0142±0.0004  0.014-0.0151 | 0.0148±0.0009  0.0134-0.0162 | 0.0158±0.0003  0.0155-0.0161 | 0.0136±0.0005  0.0128-0.0139 | 0.0161 | 0.0008±0.0008  0-0.0015 |  |
| N | 0.0439±0.001  0.0434-0.0461 | 0.0460±0.0110  0.0448-0.0475 | 0.0532±0.0014  0.0515-0.0543 | 0.0484±0.0006  0.0475-0.0488 | 0.0509 | 0.0495 | - |

**Table S2. Genetic distances of 18S rRNA between 23 freshwater fish trypanosomes, including gaps.**

|  | A | B | C | D | E | I | N |
| --- | --- | --- | --- | --- | --- | --- | --- |
| A | 0.0011±0.0011 0-0.0029 |  |  |  |  |  |  |
| B | 0.0056±0.0011 0.0044-0.0088 | 0.0023±0.0020 0-0.0064 |  |  |  |  |  |
| C | 0.0217±0.0018 0.0190-0.0248 | 0.0233±0.0013 0.0209-0.0258 | 0.0057±0.0038 0-0.0093 |  |  |  |  |
| D | 0.0162±0.0010 0.0151-0.0185 | 0.0174±0.0010 0.0156-0.0195 | 0.0128±0.0012 0.0107-0.0151 | 0.0023±0.0013 0.0005-0.0034 |  |  |  |
| E | 0.0220±0.0009 0.0214-0.0238 | 0.0235±0.0007 0.0229-0.0248 | 0.0127±0.0007 0.0116-0.0136 | 0.0172±0.0006 0.0166-0.0180 | - |  |  |
| I | 0.0215±0.0009 0.0209-0.0234 | 0.0214±0.0008 0.0209-0.0229 | 0.0202±0.0004 0.0199-0.0208 | 0.0158±0.0012 0.0146-0.0175 | 0.0194 | 0.0025±0.0025  0-0.0049 |  |
| N | 0.0417±0.0009 0.0411-0.0436 | 0.0425±0.0008 0.0416-0.0441 | 0.0538±0.0008 0.0525-0.0544 | 0.0471±0.0012 0.0460-0.0487 | 0.0534 | 0.0520 | - |

**Table S3. Genetic distances of 18S rRNA between 23 freshwater fish trypanosomes, only front fragment 1390 bp, excluding gaps. (Plus additional sequences of Ab-1-1, Sg-1 in B, Ab-2-1, Pf-1 in E, S1 in F, E1 in H, Ct-1 in O)**

|  | A | B | C | D | E | F | H | I | N | O |
| --- | --- | --- | --- | --- | --- | --- | --- | --- | --- | --- |
| A | 0.0005±0.0007  (0-0.0015) |  |  |  |  |  |  |  |  |  |
| B | 0.0064±0.0010  (0.007±0.0016)  0.0044-0.0090  (0.0044-0.0114) | 0.0018±0.0014  (0.0024±0.0016)  0-0.0040  (0-0.0067) |  |  |  |  |  |  |  |  |
| C | 0.0172±0.0021  0.0145-0.0202 | 0.0212±0.0017  (0.0219±0.0021)  0.0178-0.0237  (0.0178-0.0264) | 0.0032±0.0023  0-0.0051 |  |  |  |  |  |  |  |
| D | 0.0129±0.0010  0.0120-0.0153 | 0.0160±0.00135  (0.0167±0.0018)  0.0137-0.0185  (0.0137-0.0210) | 0.0122±0.0021  0.0097-0.0144 | 0.0010±0.0007  0-0.0014 |  |  |  |  |  |  |
| E | 0.0156±0.0006  (0.0158±0.0007)  0.0153-0.0169  (0.0153-0.0177) | 0.0189±0.00112  (0.0204±0.0018)  0.0170-0.02030  (0.0170-0.0246) | 0.0089±0.0019  (0.0084±0.0017)  0.0066-0.0105  (0.0059-0.0105) | 0.0128±0.0001  (0.0131±0.0004)  0.0128  (0.0128-0.0137) | 0.0026±0.0003  0.0022-0.0029 |  |  |  |  |  |
| F | 0.014±0.0006  0.0137-0.0154 | 0.0197±0.0017  0.0171-0.0230 | 0.0139±0.0027  0.0106-0.0161 | 0.0137±0.0001  0.0136-0.0137 | 0.0123±0.0004  0.0120-0.0129 | - |  |  |  |  |
| H | 0.0173±0.0006  0.0170-0.0187 | 0.0214±0.0017  0.0188-0.0248 | 0.0161±0.0020  0.0137-0.0177 | 0.0169±0.0001  0.0169 | 0.0144±0.0007  0.0136-0.0152 | 0.0074 | - |  |  |  |
| I | 0.0180±0.0006  0.0177-0.0194 | 0.019±0.0009  (0.0196±0.0015)  0.0178-0.0202  (0.0178-0.0228) | 0.0219±0.0070  0.0210-0.0227 | 0.0166±0.0008  0.0160-0.0176 | 0.0205±0.0004  0.0202-0.0211 | 0.0194 | 0.0211 | 0.0018±0.0018  0-0.036 |  |  |
| N | 0.0593±0.0009  0.0588-0.0613 | 0.0625±0.0012  (0.0634±0.0020)  0.0611-0.0642  (0.0611-0.0677) | 0.0733±0.0028  0.0697-0.0756 | 0.0634±0.0011  0.0626-0.0650 | 0.0683±0.0010  0.0670-0.0695 | 0.0715 | 0.0758 | 0.0677 | - |  |
| O | 0.0148±0.0006  0.0145-0.0162 | 0.0197±0.0011  (0.0204±0.0017)  0.0178-0.0211  (0.0178-0.0238) | 0.0106±0.0019  0.0082-0.0121 | 0.0137±0.0001  0.0136-0.0137 | 0.0084±0.0004  0.0082-0.0089 | 0.0153 | 0.0185 | 0.0219 | 0.0722 | - |

**Table S4. Average number of nucleotide differences K and nucleotide divergence *Pi* among intra-group, only front fragment 1390 bp. (Plus additional sequences of Ab-1-1, Sg-1 in B, Ab-2-1, Pf-1 in E, S1 in F, E1 in H, Ct-1 in O)**

|  | Average number of nucleotide differences  (K) | Nucleotide diversity (*Pi*) |
| --- | --- | --- |
| A | 0.6670 | 0.0005 |
| B | 3.3210 | 0.0024 |
| C | 4.0000 | 0.0029 |
| D | 1.3330 | 0.0010 |
| E | 3.6670 | 0.0026 |

Note: Groups of F, H, I, N, O that each contains only one isolate and are therefore not included.

**Table S5. Average number of nucleotide differences *Kxy* (above the diagonal) and nucleotide divergence *Dxy* (below the diagonal) among different populations, only front fragment 1390 bp. (Plus additional sequences of Ab-1-1, Sg-1 in B, Ab-2-1, Pf-1 in E, S1 in F, E1 in H, Ct-1 in O)**

|  | A | B | C | D | E | F | H | I | N | O |
| --- | --- | --- | --- | --- | --- | --- | --- | --- | --- | --- |
| A |  | 9.4580 | 21.3330 | 17.0000 | 20.6670 | 18.3330 | 22.3330 | 23.3330 | 66.3330 | 19.3330 |
| B | 0.0068 |  | 26.9250 | 21.7920 | 26.1250 | 25.1250 | 27.1250 | 25.2500 | 70.2500 | 26.1250 |
| C | 0.0155 | 0.0196 |  | 15.0000 | 9.5330 | 16.6000 | 19.4000 | 26.2000 | 77.0000 | 13.0000 |
| D | 0.0123 | 0.0157 | 0.0108 |  | 17.3330 | 18.0000 | 22.0000 | 21.6670 | 69.6670 | 18.0000 |
| E | 0.0149 | 0.0189 | 0.0069 | 0.0124 |  | 16.3330 | 19.0000 | 26.0000 | 73.3330 | 11.3330 |
| F | 0.0132 | 0.0181 | 0.0120 | 0.0129 | 0.0117 |  | 10.0000 | 25.0000 | 76.0000 | 20.0000 |
| H | 0.0161 | 0.0196 | 0.0140 | 0.0158 | 0.0136 | 0.0071 |  | 27.0000 | 80.0000 | 24.0000 |
| I | 0.0169 | 0.0183 | 0.0189 | 0.0156 | 0.0187 | 0.0179 | 0.0194 |  | 74.0000 | 28.0000 |
| N | 0.0480 | 0.0508 | 0.0560 | 0.0504 | 0.0531 | 0.0549 | 0.0578 | 0.0536 |  | 77.0000 |
| O | 0.0140 | 0.0189 | 0.0094 | 0.0129 | 0.0081 | 0.0143 | 0.0172 | 0.0201 | 0.0557 | - |

-: Only one representative isolate in the group.

**Table S6.** **Genetic distances of 18S rRNA between 23 freshwater fish trypanosomes, only front fragment 1390 bp, including gaps. (Plus additional sequences of Ab-1-1, Sg-1 in B, Ab-2-1, Pf-1 in E, S1 in F, E1 in H, Ct-1 in O)**

|  | A | B | C | D | E | F | H | I | N | O |
| --- | --- | --- | --- | --- | --- | --- | --- | --- | --- | --- |
| A | 0.0014±0.0013  0-0.0036 |  |  |  |  |  |  |  |  |  |
| B | 0.0071±0.0013 (0.0076±0.0017)  0.0058-0.0108  (0.0058-0.0122) | 0.0024±0.0020 (0.0031±0.0020)  0-0.0067  (0-0.0079) |  |  |  |  |  |  |  |  |
| C | 0.0291±0.0028  0.0249-0.0334 | 0.0321±0.0022 (0.0328±0.0024)  0.0278-0.0355  (0.0278-0.0369) | 0.0080±0.0053  0-0.0128 |  |  |  |  |  |  |  |
| D | 0.0202±0.0013  0.0186-0.0236 | 0.0237±0.0046 (0.0241±0.0040)  0.0200-0.0416  (0.0200-0.0416) | 0.0181±0.0020  0.0149-0.0213 | 0.0029±0.0015  0.0007-0.0043 |  |  |  |  |  |  |
| E | 0.0284±0.0010 (0.0245±0.0030)  0.0277-0.0305  (0.0214-0.0305) | 0.0311±0.0009 (0.0282±0.0028)  0.0305-0.0327  (0.0250-0.0341) | 0.0160±0.0149 (0.0169±0.0002)  0.0149-0.0170  (0.0142-0.0198) | 0.0225±0.0003 (0.0204±0.0015)  0.0220-0.0227  (0.0185-0.0227) | 0.0069±0.0029  0.0029-0.0092 |  |  |  |  |  |
| F | 0.0214±0.0010  0.0207-0.0236 | 0.0261±0.0013  0.0250-0.0289 | 0.0203±0.0024  0.0171-0.0227 | 0.0183±0.0003  0.0178-0.0185 | 0.0157±0.0006  0.0150-0.0164 | - |  |  |  |  |
| H | 0.0243±0.0010  0.0236-0.0264 | 0.0276±0.0013  0.0263-0.0300 | 0.0223±0.0017  0.0199-0.0241 | 0.0211±0.0003  0.0207-0.0214 | 0.0176±0.0003  0.0171-0.0178 | 0.0071 | - |  |  |  |
| I | 0.0278±0.0010  0.0207-0.0299 | 0.0284±0.0009 (0.0289±0.0013)  0.0277-0.0299  (0.0277-0.0313) | 0.0278±0.0007  0.0269-0.0290 | 0.0218±0.0012  0.0206-0.0234 | 0.0272±0.0004  0.0269-0.0278 | 0.0220 | 0.0242 | 0.0036±0.0036  0-0.0071 |  |  |
| N | 0.0517±0.0010  0.0510-0.0539 | 0.0536±0.0010 (0.0542±0.0014)  0.0524-0.0553  (0.0524-0.0568) | 0.0697±0.0015  0.0674-0.0709 | 0.0604±0.0012  0.0592-0.0621 | 0.0652±0.0020  0.0634-0.0680 | 0.0663 | 0.0691 | 0.0675 | - |  |
| O | 0.0193±0.0010  0.0186-0.0215 | 0.0241±0.0013  0.0229-0.0265 | 0.0202±0.0022  0.0170-0.0220 | 0.0197±0.0003  0.0193-0.0200 | 0.0124±0.0029  0.0100-0.0164 | 0.0186 | 0.0214 | 0.0291 | 0.0636 | - |

**Table S7. Genetic distances of 18S rRNA between 23 freshwater fish trypanosomes, only back fragment 1237bp, excluding gaps. (Plus additional sequences of Ct-2 in A, Ab-1-2, Ab2-2, Sg-2 in E, Pf-2 in M)**

|  | A | B | C | D | E | I | M | N |
| --- | --- | --- | --- | --- | --- | --- | --- | --- |
| A | 0.0006±0.0008  (0.0005±0.0008)  0-0.0017 |  |  |  |  |  |  |  |
| B | 0.0017±0.0009  (0.0016±0.0009)  0.0008-0.0042 | 0.0010±0.0007  0-0.0025 |  |  |  |  |  |  |
| C | 0.0164±0.0009  (0.0164±0.0008)  0.0157-0.0186 | 0.0174±0.0010  0.0157-0.0196 | 0.0010±0.0008  0-0.0016 |  |  |  |  |  |
| D | 0.0136±0.0008  (0.0136±0.0079)  0.0130-0.0158 | 0.0146±0.0010  0.0130-0.0168 | 0.0095±0.00039  0.0093-0.0102 | 0.0016±0.0012  0-0.0025 |  |  |  |  |
| E | 0.0152±0.0007  (0.0180±0.0019)  0.0149-0.0169 | 0.0165±0.0007  (0.0191±0.0019)  0.0158-0.0178  (0.0158-0.0226) | 0.0079±0.0017  (0.0092±0.0020)  0.0059-0.0093  (0.0059-0.0119) | 0.0127±0.0005  (0.0140±0.0011)  0.0120-0.0130  (0.0120-0.0156) | 0 (0.0041±0.0011)  0 (0.0025±0.0058) |  |  |  |
| I | 0.0181±0.0008  (0.0181±0.0007)  0.0178-0.0198 | 0.0190±0.0009  0.0177-0.0207 | 0.0208±0.0005  0.0204-0.0214 | 0.0170±0.0005  0.0166-0.0176 | 0.0189±0.0010  0.0175  (0.0175-0.0202) | 0.0013±0.0013  0-0.0025 |  |  |
| M | 0.0954±0.0011  0.0949-0.0981 | 0.0972±0.0012  0.0963-0.0996 | 0.1118±0.0015  0.1101-0.1135 | 0.1061±0.0009  0.1055-0.1074 | 0.1086±0.0020  0.1062  (0.1062-0.1118) | 0.1055 | - |  |
| N | 0.0510±0.0010  (0.0510±0.0009)  0.0505-0.0533 | 0.0521±0.0012 0.0504-0.0542 | 0.0649±0.0001  0.0647-0.0650 | 0.0599±0.0007  0.0594-0.0610 | 0.0625±0.0008  0.0613  (0.0613-0.0632) | 0.0617 | 0.0427 | - |

**Table S8. Average number of nucleotide differences K and nucleotide divergence *Pi* among intra-group, only back fragment 1237bp. (Plus additional sequences of Ct-2 in A, Ab-1-2, Ab2-2, Sg-2 in E, Pf-2 in M)**

|  | Average number of nucleotide differences (K) | Nucleotide diversity (*Pi*) |
| --- | --- | --- |
| A | 0.5710 | 0.0005 |
| B | 1.2000 | 0.0010 |
| C | 1.2000 | 0.0010 |
| D | 2.0000 | 0.0016 |
| E | 5.0000 | 0.0041 |

Note: Groups of I, M, N that each contains only one isolate and are therefore not included.

**Table S9. Average number of nucleotide differences *Kxy* (above the diagonal) and nucleotide divergence *Dxy* (below the diagonal) among different populations,** **only back fragment 1237bp. (Plus additional sequences of Ct-2 in A, Ab-1-2, Ab2-2, Sg-2 in E, Pf-2 in M)**

|  | A | B | C | D | E | I | M | N |
| --- | --- | --- | --- | --- | --- | --- | --- | --- |
| A |  | 1.9520 | 18.6860 | 15.6190 | 20.2860 | 20.2860 | 86.2860 | 51.2860 |
| B | 0.0016 |  | 19.7330 | 16.6670 | 21.4170 | 21.3330 | 87.3330 | 52.3330 |
| C | 0.0153 | 0.0161 |  | 11.0670 | 9.7000 | 23.0000 | 95.4000 | 61.4000 |
| D | 0.0128 | 0.0136 | 0.0090 |  | 16.1670 | 19.3330 | 93.3330 | 58.3330 |
| E | 0.0166 | 0.0175 | 0.0079 | 0.0131 |  | 21.5000 | 94.0000 | 59.5000 |
| I | 0.0166 | 0.0174 | 0.0187 | 0.0157 | 0.0175 |  | 93.0000 | 60.0000 |
| M | 0.0707 | 0.0715 | 0.0779 | 0.0763 | 0.0769 | 0.0763 |  | 45.0000 |
| N | 0.0421 | 0.0429 | 0.0503 | 0.0478 | 0.0487 | 0.0493 | 0.0368 | - |

-: Only one representative isolate in the group.

**Table S10. Genetic distances of 18S rRNA between 23 freshwater fish trypanosomes, only back fragment 1237bp, including gaps. (Plus additional sequences of Ct-2 in A, Ab-1-2, Ab2-2, Sg-2 in E, Pf-2 in M)**

|  | A | B | C | D | E | I | M | N |
| --- | --- | --- | --- | --- | --- | --- | --- | --- |
| A | 0.0019±0.0014  (0.0016±0.0014)  0-0.0041 (0-0.0041) |  |  |  |  |  |  |  |
| B | 0.0033±0.0013  (0.0031±0.0013) 0.0016-0.0065 (0.0016-0.0065) | 0.0018±0.0015  0-0.0049 |  |  |  |  |  |  |
| C | 0.0280±0.0013  (0.0278±0.0013) 0.0258-0.0313 (0.0258-0.0313) | 0.0274±0.0010  0.0257-0.0297 | 0.0047±0.0033  0-0.0080 |  |  |  |  |  |
| D | 0.0203±0.0013  (0.0202±0.0012)  0.0186-0.0234 (0.0186-0.0234) | 0.0198±0.0009  0.0186-0.0218 | 0.0136±0.0013  0.0113-0.0161 | 0.0038±0.0021  0.0008-0.0057 |  |  |  |  |
| E | 0.0275±0.0011  (0.0292±0.0016)  0.0266-0.0298 (0.0266-0.0330) | 0.0274±0.0009  (0.0290±0.0013) 0.0266-0.0289 (0.0266-0.0314) | 0.0148±0.0004  (0.0154±0.0008) 0.0144-0.0152 (0.0144-0.0168) | 0.0188±0.0010  (0.0191±0.0012) 0.0178-0.0201 (0.0178-0.0217) | 0  (0.0044±0.0008)  0 (0.0032-0.0056) |  |  |  |
| I | 0.0276±0.0011  (0.0274±0.0011) 0.0266-0.0298 (0.0266-0.0298) | 0.0272±0.0006  0.0266-0.0283 | 0.0265±0.0005  0.0257-0.0273 | 0.0205±0.0020  0.0187-0.0233 | 0.0239±0.0007  0.0233-0.0249 | 0.0041±0.0041  0-0.0081 |  |  |
| M | 0.0656±0.0011  (0.0655±0.0011) 0.0648-0.0681 (0.0648-0.0681) | 0.0654±0.0006  0.0649-0.0665 | 0.0804±0.0004  0.0801-0.0810 | 0.0738±0.0019  0.0721-0.0765 | 0.0809±0.0010  0.0801-0.0825 | 0.0795 | - |  |
| N | 0.0472±0.0012  (0.0471±0.0011)  0.0464-0.0496 (0.0464-0.0496) | 0.0469±0.0006  0.0464-0.0481 | 0.0631±0.0004  0.6275-0.6361 | 0.0563±0.0019  0.0546-0.0590 | 0.0632±0.0004  0.0628-0.0636 | 0.0629 | 0.0261 | - |

**Table S11.** **Genetic distances of 18S rRNA between 23 freshwater fish trypanosomes, middle fragment 1489bp, excluding gaps. (From 278bp to 1766bp). (****Plus additional sequences of *Trypanosoma granulosum*_Portugal in G)**

|  | A | B | C | D | E | G | I | N |
| --- | --- | --- | --- | --- | --- | --- | --- | --- |
| A | 0.0007±0.0010  0-0.0021 |  |  |  |  |  |  |  |
| B | 0.0020±0.0009  0.0014-0.0048 | 0.0006±0.0006  0-0.0014 |  |  |  |  |  |  |
| C | 0.0129±0.0009  0.0120-0.0151 | 0.0129±0.0007  0.0121-0.0143 | 0.0004±0.0003  0-0.0007 |  |  |  |  |  |
| D | 0.0092±0.0011  0.0084-0.0121 | 0.0093±0.0009  0.0084-0.0114 | 0.0068±0.0005  0.0062-0.0076 | 0.0009±0.0006  0-0.0013 |  |  |  |  |
| E | 0.0110±0.0009  0.0106-0.0128 | 0.0110±0.0006  0.0106-0.0121 | 0.0054±0.0010  0.0041-0.0062 | 0.0069±0.0001  0.0069 | - |  |  |  |
| G | 0.0140±0.0009  0.0136-0.0160 | 0.0141±0.0006  0.0137-0.0152 | 0.0104±0.0004  0.0099-0.0107 | 0.0084±0.0001  0.0084 | 0.0091 | - |  |  |
| I | 0.0125±0.0009  0.0121-0.0144 | 0.0125±0.0006  0.0121-0.0136 | 0.0155±0.0003  0.0151-0.0157 | 0.0111±0.0010  0.0106-0.0121 | 0.0135 | 0.0167 | 0.0010±0.0010  0-0.0020 |  |
| N | 0.0503-0.0538 | 0.0509±0.0008  0.0503-0.0525 | 0.0608±0.0004  0.0602-0.0611 | 0.0556±0.0010  0.0549-0.0571 | 0.0569 | 0.0583 | 0.0586 | - |

**Table S12.** **Genetic distances of 18S rRNA between 23 freshwater fish trypanosomes, middle fragment 633bp, excluding gaps. (From 745bp to 1377bp). (Plus additional sequences of abel in J, L460 in K and SSH2 in L)**

|  | A | B | C | D | E | I | J | K | L | N |
| --- | --- | --- | --- | --- | --- | --- | --- | --- | --- | --- |
| A | 0.0005±0.0008  0-0.0016 |  |  |  |  |  |  |  |  |  |
| B | 0.0024±0.0010  0.0016-0.0049 | 0.0009±0.0008  0-0.0016 |  |  |  |  |  |  |  |  |
| C | 0.0263±0.0007  0.0260-0.0279 | 0.0286±0.0009  0.0279-0.0299 | 0.0009±0.0008  0-0.0016 |  |  |  |  |  |  |  |
| D | 0.0202±0.0019  0.0187-0.0243 | 0.0224±0.0020  0.0206-0.0263 | 0.0135±0.0007  0.0131-0.0149 | 0.0021±0.0015  0-0.0032 |  |  |  |  |  |  |
| E | 0.0209±0.0007  0.0206-0.0225 | 0.0232±0.0009  0.0226-0.0244 | 0.0094±0.0024  0.0064-0.0114 | 0.0132±0.0001  0.0131-0.0132 | - |  |  |  |  |  |
| I | 0.0284±0.0007  0.0281-0.0301 | 0.0307±0.0010  0.0301-0.0321 | 0.0353±0.0001  0.0353-0.0355 | 0.0272±0.0018  0.0259-0.0298 | 0.0295 | 0.0024±0.0024  0-0.0048 |  |  |  |  |
| J | 0.0775±0.0012  0.0769-0.0801 | 0.0801±0.0015  0.0791-0.0823 | 0.1191±0.0024  0.1159-0.1211 | 0.1096±0.0013  0.1087-0.1114 | 0.1069 | 0.1116 | - |  |  |  |
| K | 0.0934±0.0012  0.0928-0.0961 | 0.0961±0.0016  0.0950-0.0984 | 0.1367±0.0038  0.1318-0.1398 | 0.1358±0.0032  0.1335-0.1404 | 0.1275 | 0.1328 | 0.0188 | - |  |  |
| L | 0.0931±0.0012  0.0925-0.0958 | 0.0958±0.0018  0.0947-0.0980 | 0.1406±0.0039  0.1357-0.1438 | 0.1342±0.0013  0.1332-0.1361 | 0.1312 | 0.1364 | 0.0082 | 0.0098 | - |  |
| N | 0.0840±0.0011  0.8350-0.0863 | 0.0871±0.0014  0.0861-0.0890 | 0.1089±0.0001  0.1088-0.1091 | 0.0998±0.0029  0.0978-0.1040 | 0.0983 | 0.1085 | 0.1369 | 0.1650 | 0.1646 | - |

**Table S13. Average number of nucleotide differences K and nucleotide divergence *Pi* among intra-group, middle fragment 1489bp (From 278bp to 1766bp). (Plus additional sequences of *Trypanosoma granulosum*_Portugal in G)**

|  | Average number of nucleotide differences (K) | Nucleotide diversity (*Pi*) |
| --- | --- | --- |
| A | 1.0000 | 0.0007 |
| B | 0.8670 | 0.0006 |
| C | 0.6000 | 0.0004 |
| D | 1.3330 | 0.0009 |

Note: Groups E, G, I, N each contains only one isolate and are therefore not included.

**Table S14. Average number of nucleotide differences K and nucleotide divergence *Pi* among intra-group, middle fragment 633bp (From 745bp to 1377bp). (Plus additional sequences of abel in J, L460 in K and SSH2 in L)**

|  | Average number of nucleotide differences (K) | Nucleotide diversity (*Pi*) |
| --- | --- | --- |
| A | 0.3330 | 0.0005 |
| B | 0.5330 | 0.0008 |
| C | 0.6000 | 0.0009 |
| D | 1.3330 | 0.0021 |

Note: Groups E, I, J, K, L, N each contains only one isolate and are therefore not included.

**Table S15. Average number of nucleotide differences *Kxy* (above the diagonal) and nucleotide divergence *Dxy* (below the diagonal) among different populations, middle fragment 1489bp (From 278bp to 1766bp). (Plus additional sequences of *Trypanosoma granulosum*_Portugal in G)**

|  | A | B | C | D | E | G | I | N |
| --- | --- | --- | --- | --- | --- | --- | --- | --- |
| A |  | 3.0000 | 17.5000 | 13.1670 | 15.5000 | 19.5000 | 17.5000 | 61.5000 |
| B | 0.0020 |  | 17.5000 | 13.1670 | 15.5000 | 19.5000 | 17.5000 | 61.5000 |
| C | 0.0118 | 0.0118 |  | 9.0000 | 6.6000 | 14.0000 | 20.6000 | 70.0000 |
| D | 0.0089 | 0.0089 | 0.0060 |  | 10.0000 | 12.0000 | 15.6670 | 65.6670 |
| E | 0.0104 | 0.0104 | 0.0044 | 0.0067 |  | 13.0000 | 19.0000 | 67.0000 |
| G | 0.0133 | 0.0133 | 0.0095 | 0.0081 | 0.0088 |  | 23.0000 | 68.0000 |
| I | 0.0118 | 0.0118 | 0.0138 | 0.0105 | 0.0127 | 0.0156 |  | 69.0000 |
| N | 0.0415 | 0.0414 | 0.0472 | 0.0442 | 0.0452 | 0.0463 | 0.0466 | - |

-: Only one representative isolate in the group.

**Table S16. Average number of nucleotide differences *Kxy* (above the diagonal) and nucleotide divergence *Dxy* (below the diagonal) among different populations, middle fragment 633bp (From 745bp to 1377bp). (Plus additional sequences of abel in J, L460 in K and SSH2 in L)**

|  | A | B | C | D | E | I | J | K | L | N |
| --- | --- | --- | --- | --- | --- | --- | --- | --- | --- | --- |
| A |  | 1.5000 | 15.1670 | 11.8330 | 12.1670 | 16.1670 | 37.1670 | 44.1670 | 44.1670 | 41.1670 |
| B | 0.0024 |  | 16.3330 | 13.0000 | 13.3330 | 17.3330 | 38.3330 | 45.3330 | 45.3330 | 42.3330 |
| C | 0.0241 | 0.0259 |  | 8.0000 | 4.6000 | 19.6000 | 52.6000 | 59.6000 | 60.6000 | 50.0000 |
| D | 0.0188 | 0.0205 | 0.0126 |  | 8.0000 | 15.6670 | 49.3330 | 59.6670 | 59.3330 | 46.6670 |
| E | 0.0193 | 0.0211 | 0.0072 | 0.0126 |  | 17.0000 | 49.0000 | 58.0000 | 59.0000 | 46.0000 |
| I | 0.0258 | 0.0276 | 0.0309 | 0.0247 | 0.0267 |  | 50.0000 | 59.0000 | 60.0000 | 50.0000 |
| J | 0.0601 | 0.0618 | 0.0842 | 0.0792 | 0.0780 | 0.0804 |  | 11.0000 | 5.0000 | 58.0000 |
| K | 0.0703 | 0.0720 | 0.0936 | 0.0938 | 0.0903 | 0.0929 | 0.0175 |  | 6.0000 | 67.0000 |
| L | 0.0706 | 0.07220 | 0.0956 | 0.0936 | 0.0923 | 0.0948 | 0.0079 | 0.0094 |  | 67.0000 |
| N | 0.0657 | 0.0673 | 0.0795 | 0.0742 | 0.0733 | 0.0799 | 0.0940 | 0.1069 | 0.1072 | - |

-: Only one representative isolate in the group.

**Table S17. Genetic distances of 18S rRNA between 23 freshwater fish trypanosomes, middle fragment 1489bp (From 278bp to 1766bp), including gaps. (Plus additional sequences of *Trypanosoma granulosum*_Portugal in G)**

|  | A | B | C | D | E | G | I | N |
| --- | --- | --- | --- | --- | --- | --- | --- | --- |
| A | 0.0011±0.0009  0-0.0027 |  |  |  |  |  |  |  |
| B | 0.0025±0.0010  0.0013-0.0054 | 0.0010±0.0008  0-0.0027 |  |  |  |  |  |  |
| C | 0.0226±0.0012  0.0206-0.0252 | 0.0224±0.0013  0.0200-0.0252 | 0.0042±0.0031  0-0.0073 |  |  |  |  |  |
| D | 0.0141±0.0011  0.0127-0.0167 | 0.0140±0.0011  0.0127-0.0167 | 0.0120±0.0012  0.0100-0.0139 | 0.0027±0.0014  0.0007-0.0040 |  |  |  |  |
| E | 0.0218±0.0007  0.0213-0.0233 | 0.0218±0.0007  0.0213-0.0233 | 0.0118±0.0005  0.0113-0.0126 | 0.0150±0.0003  0.0146-0.0153 | - |  |  |  |
| G | 0.0233±0.0007  0.0228-0.0248 | 0.0232±0.0007  0.0227-0.0248 | 0.0260±0.0010  0.0246-0.0272 | 0.0205±0.0003  0.0200-0.0207 | 0.0246 | - |  |  |
| I | 0.0199±0.0007  0.0193-0.0213 | 0.0199±0.0007  0.0193-0.0231 | 0.0216±0.0007  0.0206-0.0225 | 0.0157±0.0011  0.0146-0.0173 | 0.0199 | 0.0287 | 0.0034±0.0034  0-0.0067 |  |
| N | 0.0435±0.0008  0.0430-0.0450 | 0.0435±0.0007  0.0430-0.0450 | 0.0578±0.0004  0.0571-0.0584 | 0.0511±0.0011  0.0500-0.0527 | 0.0571 | 0.0569 | 0.0566 | - |

**Table S18. Genetic distances of 18S rRNA between 23 freshwater fish trypanosomes, middle fragment 633bp, including gaps. (From 745bp to 1377bp). (Plus additional sequences of abel in J, L460 in K and SSH2 in L)**

|  | A | B | C | D | E | I | J | K | L | N |
| --- | --- | --- | --- | --- | --- | --- | --- | --- | --- | --- |
| A | 0.0016±0.0010  0-0.0032 |  |  |  |  |  |  |  |  |  |
| B | 0.0032±0.0011  0.0016-0.0047 | 0.0014±0.0010  0-0.0032 |  |  |  |  |  |  |  |  |
| C | 0.0473±0.0023  0.0432-0.0507 | 0.0483±0.0024  0.0432-0.0522 | 0.0080±0.0057  0-0.0138 |  |  |  |  |  |  |  |
| D | 0.0309±0.0021  0.0280-0.0342 | 0.0322±0.0021  0.0296-0.0358 | 0.0247±0.0027  0.0201-0.0291 | 0.0062±0.0034  0.0016-0.0093 |  |  |  |  |  |  |
| E | 0.0440±0.0008  0.0432-0.0448 | 0.0455±0.0008  0.0448-0.0463 | 0.0227±0.0011  0.0215-0.0245 | 0.0302±0.0007  0.0292-0.0307 | - |  |  |  |  |  |
| I | 0.0442±0.0008  0.0434-0.0450 | 0.0457±0.0008  0.0450-0.0465 | 0.0469±0.0015  0.0445-0.0489 | 0.0365±0.0026  0.0340-0.0401 | 0.0415 | 0.0078±0.0078  0-0.0155 |  |  |  |  |
| J | 0.0763±0.0008  0.0755-0.0770 | 0.0778±0.0008  0.0770-0.0786 | 0.1039±0.0014  0.1017-0.1058 | 0.0958±0.0007  0.0949-0.0963 | 0.0988 | 0.1022 | - |  |  |  |
| K | 0.0777±0.0008  0.0769-0.0785 | 0.0792±0.0008  0.0785-0.0800 | 0.0967±0.0007  0.0960-0.0976 | 0.0909±0.0018  0.0890-0.0933 | 0.0908 | 0.1009 | 0.0356 | - |  |  |
| L | 0.0783±0.0008  0.0775-0.0791 | 0.0798±0.0008  0.0791-0.0806 | 0.1008±0.0012  0.0996-0.1028 | 0.0939±0.0013  0.0925-0.0955 | 0.0955 | 0.1060 | 0.0250 | 0.0170 | - |  |
| N | 0.0702±0.0008  0.0694-0.0710 | 0.0718±0.0008  0.0710-0.0726 | 0.1016±0.0009  0.1002-0.1029 | 0.0896±0.0027  0.0871-0.0933 | 0.0985 | 0.1020 | 0.1078 | 0.1108 | 0.1085 | - |


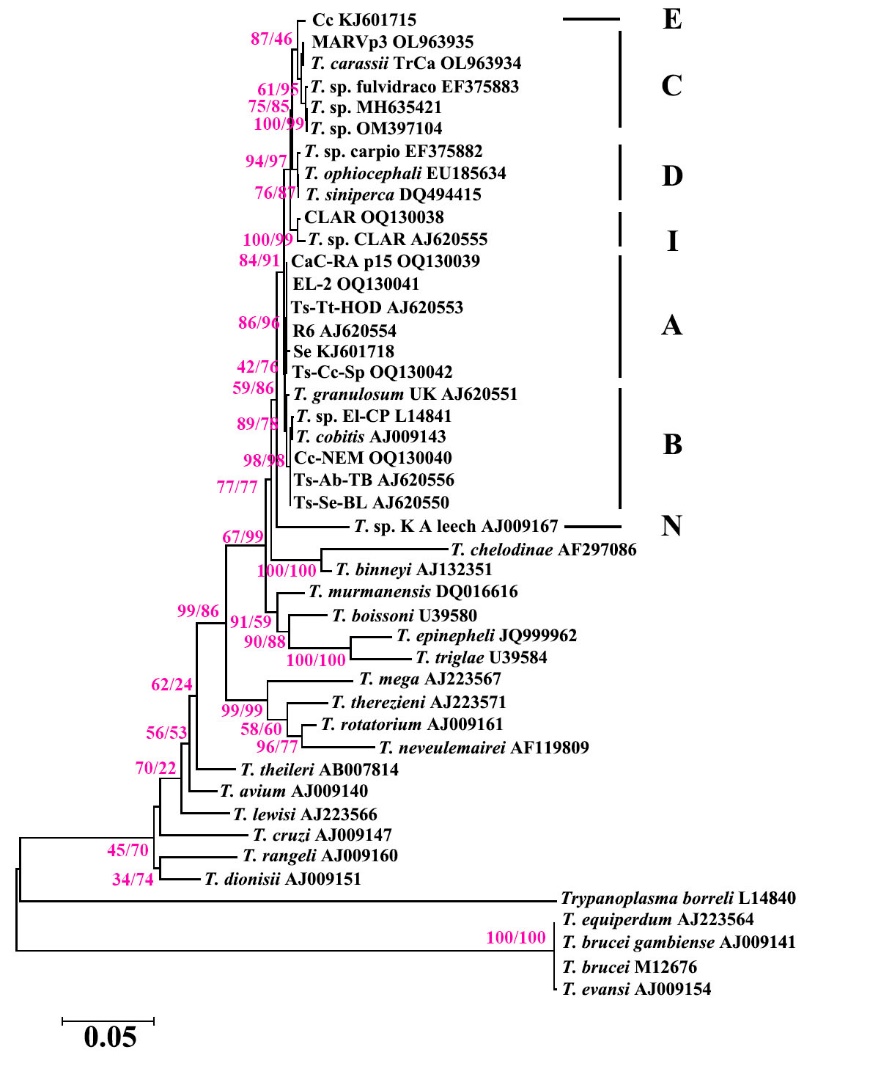


Fig. S1. 18S rRNA (2 kb)-based phylogenetic tree of trypanosomes. Bootstrap values (of neighbor-joining / maximum likelihood methods) shown at the nodes were counted with 1000 repetitions. Bar represents 0.05 substitutions per site.





Fig.S2. 18S rRNA gene amplicons and corresponding maximum genetic distance between different OTUs. Correlation coefficient excluding gaps of 2kb fragment is considered as reference (1.00). A schematic 18S rRNA gene and shorter amplicons of freshwater fish trypanosomes, with variable (V) and conserved (U) regions indicated.


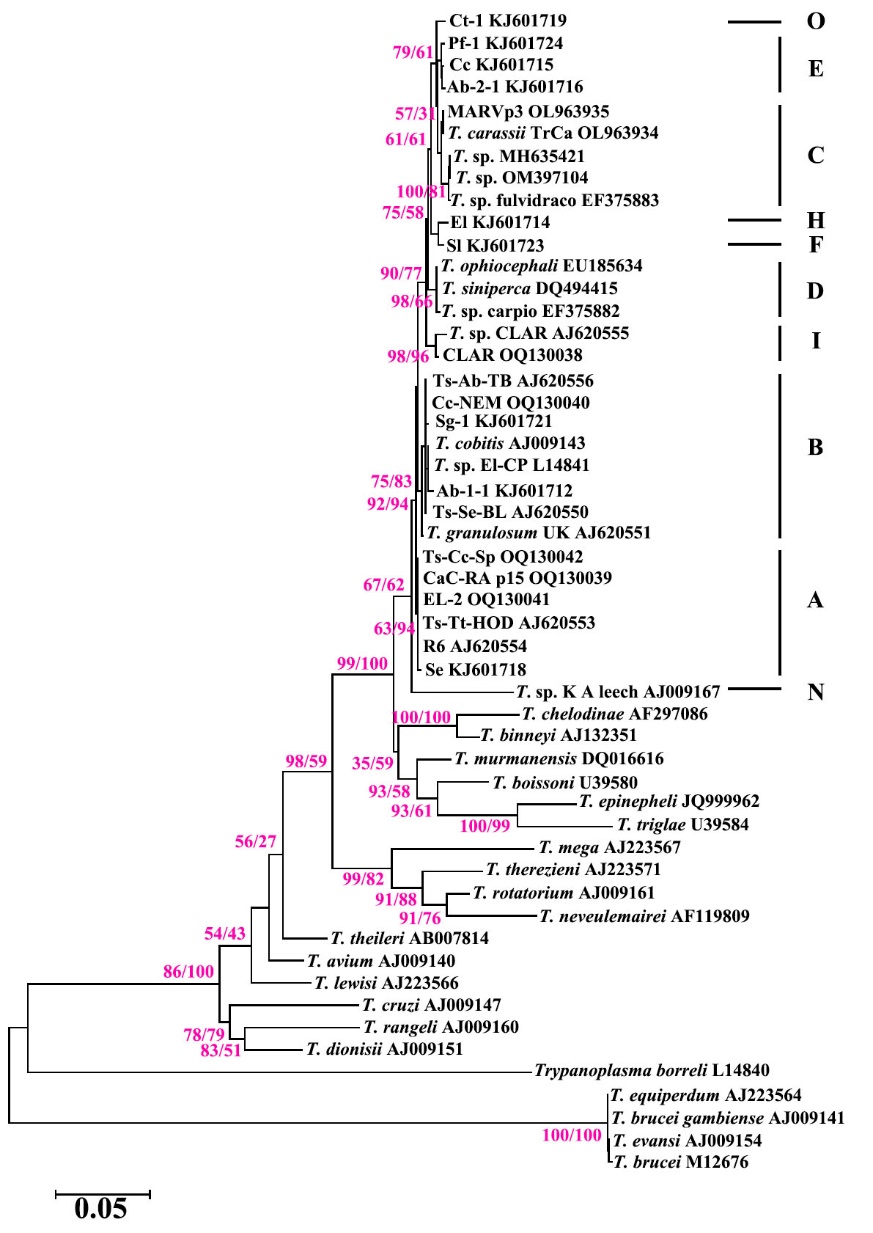


Fig. S3. 18S rRNA (1.4 kb)-based phylogenetic tree of trypanosomes. Bootstrap values (of neighbor-joining / maximum likelihood methods) shown at the nodes were counted with 1000 repetitions. Bar represents 0.05 substitutions per site.


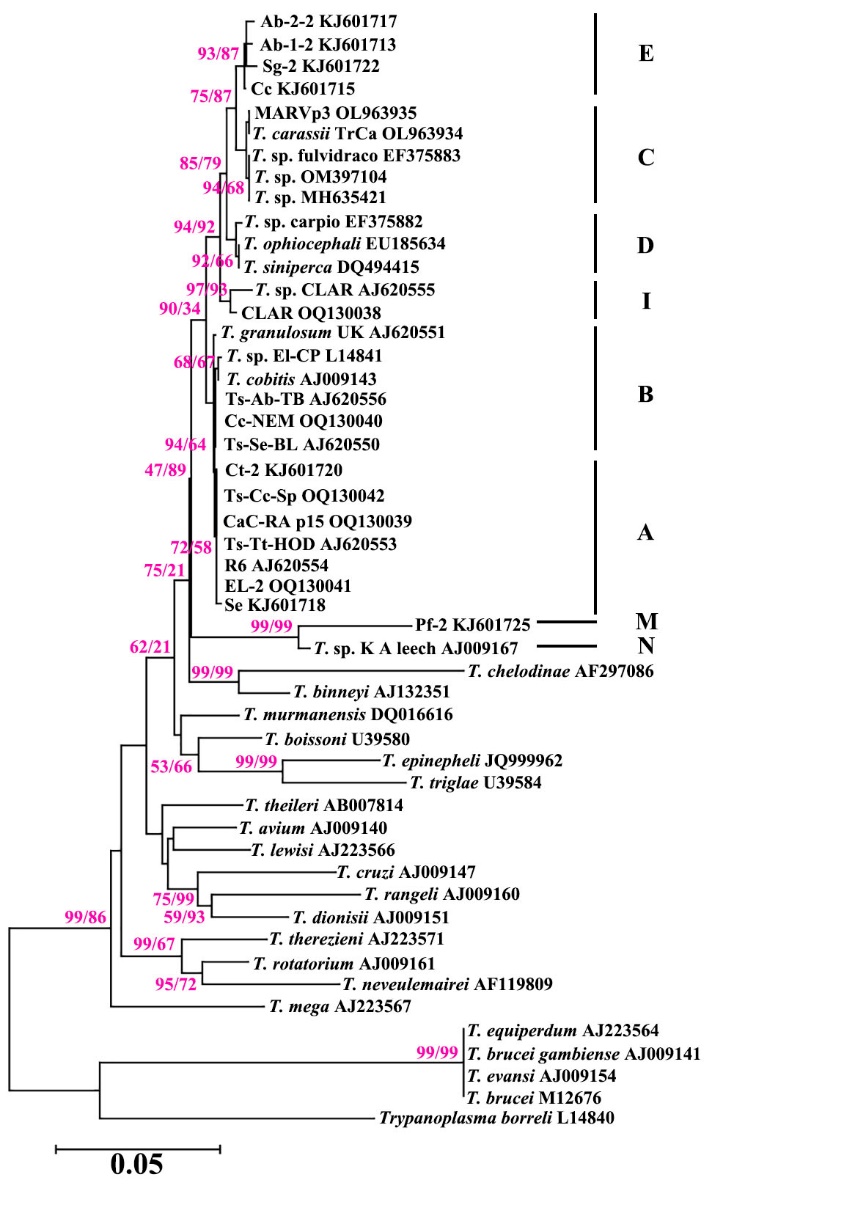
Fig. S4. 18S rRNA (1.2 kb)-based phylogenetic tree of trypanosomes. Bootstrap values (of neighbor-joining / maximum likelihood methods) shown at the nodes were counted with 1000 repetitions. Bar represents 0.05 substitutions per site.


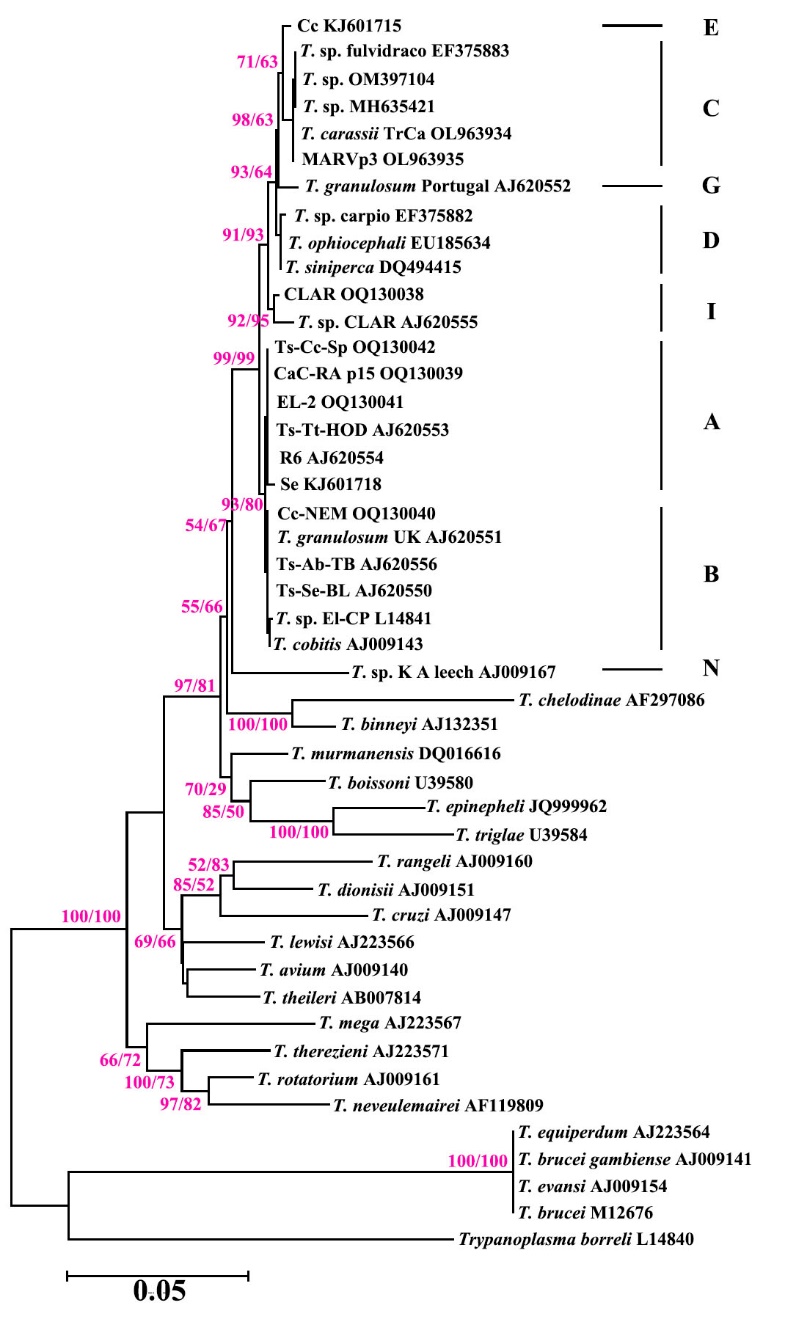
Fig. S5. 18S rRNA (1.5kb)-based phylogenetic tree of trypanosomes. Bootstrap values (of neighbor-joining / maximum likelihood methods) shown at the nodes were counted with 1000 repetitions. Bar represents 0.05 substitutions per site.


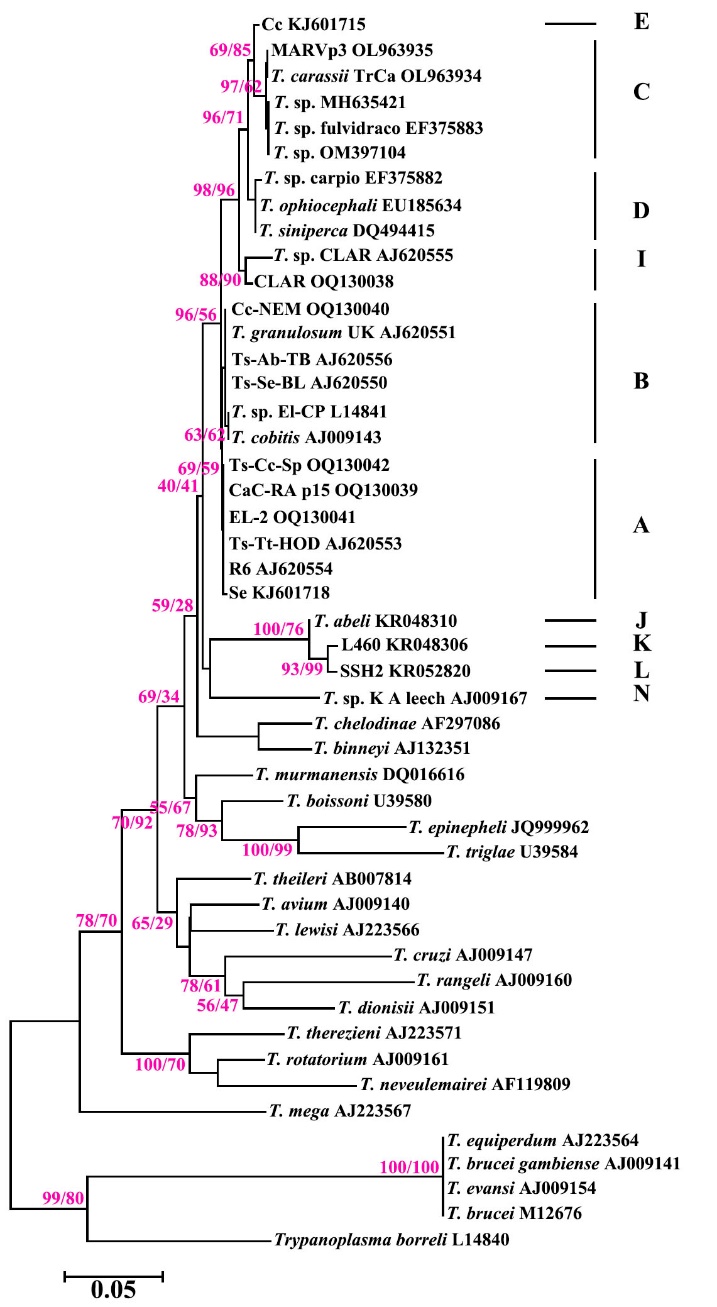


Fig. S6. 18S rRNA (0.6 kb)-based phylogenetic tree of trypanosomes. Bootstrap values (of neighbor-joining / maximum likelihood methods) shown at the nodes were counted with 1000 repetitions. Bar represents 0.05 substitutions per site.


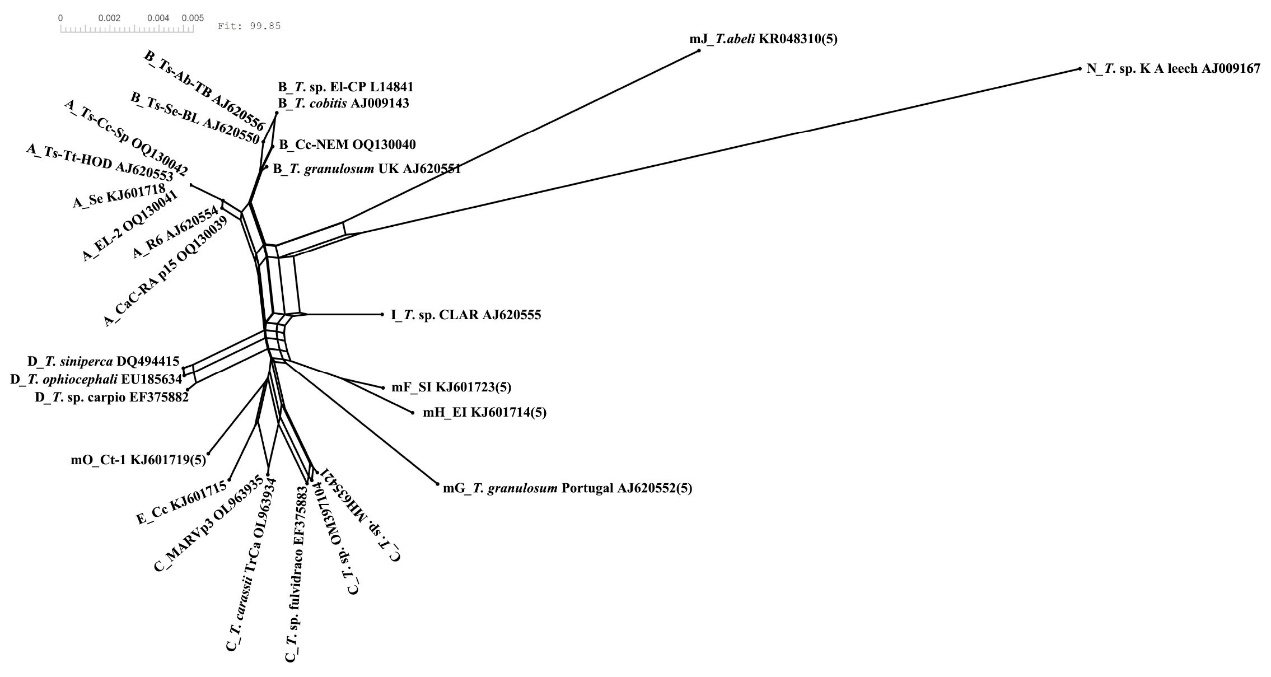


Fig. S7. Network genealogies of 18S rRNA gene sequences using the Neighbour-Net method.


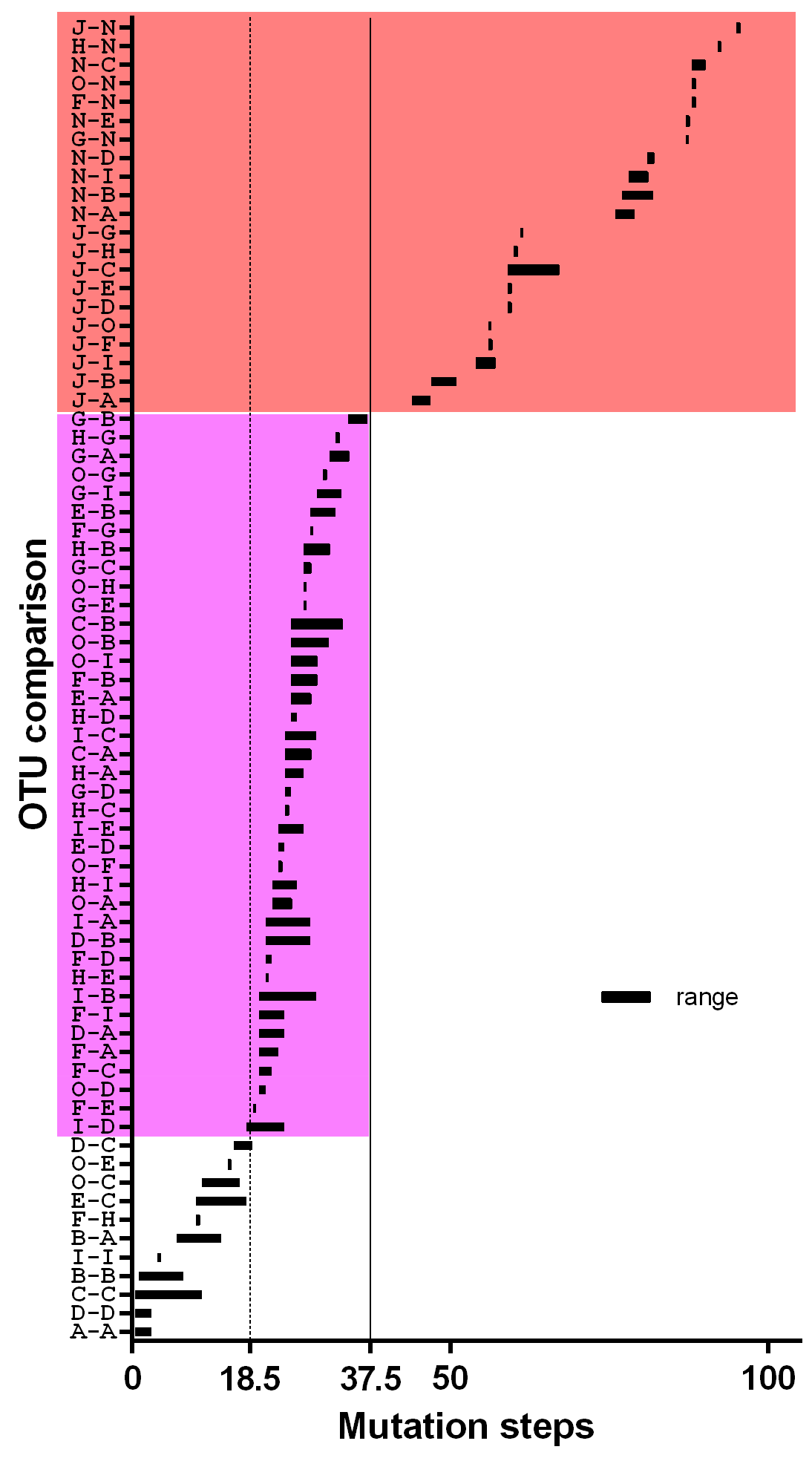


Fig. S8. Mutation steps between different OTUs. Purple indicates the mutation steps between 19-37. Orange indicates the mutation steps of 38 or greater.


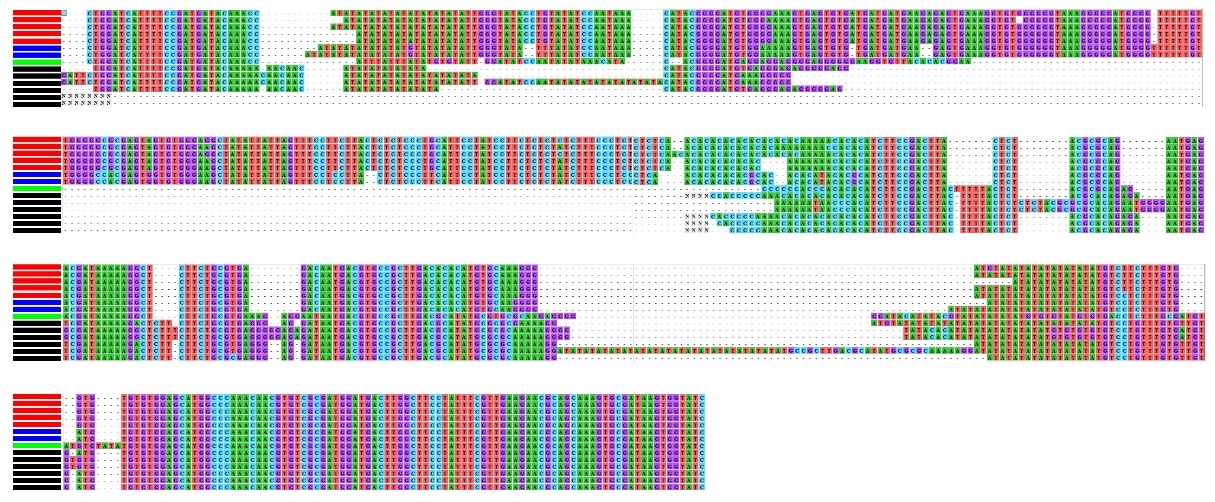
Fig. S9. ITS1 sequence of freshwater fish trypanosomes. The full length varies from 347-499bp, divided into four color section, each section showing 248 nucleotides. Nucleotides are displayed as color blocks of green (A), red (T), blue (C), violet (G) gray (unknown) and white (gaps). OTUs are indicated as color lines on the left, with OTU A in red, OTU B in blue, OTU C in black and OTU I in green.





Fig. S10. Host information of freshwater fish trypanosomes with different OTUs. The phylogenetic tree of fish is adapted from Betancur-R et al., 2017.


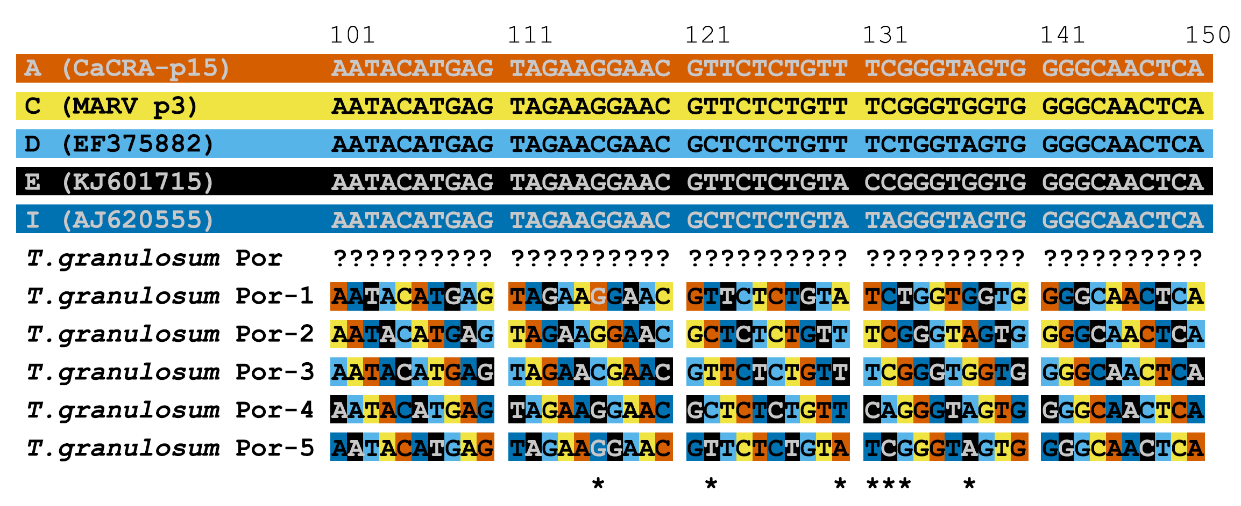
Fig. S11. An example of generation a set hypothetic sequences for Parsimony (TCS) and neighbor networks of haplotype diversity analysis.

Reference:

Betancur-R R, Wiley EO, Arratia G, Acero A, Bailly N, Miya M, Lecointre G, Ortí G (2017) Phylogenetic classification of bony fishes. BMC Evol Biol 17:162
